# Accuracy of Novice Raters for Esophageal Motility Classifications Using Functional Lumen Imaging Probe Panometry

**DOI:** 10.1111/nmo.70386

**Published:** 2026-07-05

**Authors:** Cayla E. Bales, Anthony J. Kang, Jacob M. Schauer, Keerthana Chakka, Benjamin M. Moy, Chiagoziem Ogbonna, Amanda Pirola, Aidan Smires, Mark A. Solinski, Rena Yadlapati, John E. Pandolfino, Dustin A. Carlson

**Affiliations:** ^1^ Kenneth C. Griffin Esophageal Center of Northwestern Medicine, Department of Medicine, Division of Gastroenterology and Hepatology, Feinberg School of Medicine Northwestern University Chicago Illinois USA; ^2^ Division of Biostatistics and Informatics, Department of Preventive Medicine, Feinberg School of Medicine Northwestern University Chicago Illinois USA; ^3^ Division of Gastroenterology University of California San Diego San Diego California USA

**Keywords:** achalasia, dysphagia, endoscopy, impedance, motility

## Abstract

**Introduction:**

Functional lumen imaging probe (FLIP) Panometry classifies esophageal motility using esophagogastric junction (EGJ) opening and esophageal contractile patterns. A previous study evaluated FLIP Panometry interpretation among esophageal specialists (which demonstrated an average accuracy of 78% for FLIP motility diagnoses), but the interpretation performance of nonexperts remains unknown. This study aimed to assess the accuracy and interrater agreement of novice raters using FLIP Panometry v1.0 and the novel v2.0 (Dallas Consensus) motility classification schemes.

**Methods:**

Eight non‐gastroenterology trainees without previous FLIP or esophageal motility experience interpreted 40 FLIP studies. Brief training included a video tutorial and review of 10 practice studies with an esophageal specialist. Accuracy was assessed using percent agreement with the reference standard assigned by two experienced raters. Interrater agreement was assessed using intraclass correlation coefficient (ICC) and Fleiss' kappa.

**Results:**

Accuracy of motility diagnosis was mean (95% CI) 0.73 (0.68–0.78) for v2.0 and 0.78 (0.73–0.82) for v1.0. Accuracies ≥ 78% were achieved by four raters for v2.0 and five for v1.0, while other raters' accuracies ranged from 55% to 75%. Accuracy for EGJ opening classification was 0.80 (0.75–0.84) and for contractile response pattern was 0.78 (0.74–0.83) for v2.0 and 0.78 (0.74–0.83) for v1.0. Moderate‐to‐good interrater agreement was observed (ICC 0.80–0.83; kappa 0.55–0.61).

**Conclusion:**

Following brief training, some novice raters achieved accuracy akin to esophageal motility specialists when interpreting FLIP Panometry motility studies. While variation in accuracy highlights the need for improved and adequate training methods for esophageal motility interpretation, accurate FLIP Panometry interpretation was achievable among nonspecialists, supporting feasibility for broad use.

## Key Points

- FLIP panometry is used to diagnose esophageal motility disorders. Motility experts can interpret FLIP panometry with high levels of accuracy and interrater agreement.
- After brief training, some novice raters can interpret FLIP panometry similarly to experts. These results support feasibility for use of FLIP in broad, general gastroenterology practice.

## Plain Language Summary

Gastroenterologists use a tool called FLIP Panometry to diagnose swallowing disorders by measuring how well the esophagus opens and contracts. Until now, only motility specialists had been tested on how accurately they could interpret FLIP results. This study tested eight trainees with no prior experience reading FLIP (nor esophageal manometry). After brief training, which included a video tutorial and review of 10 practice cases, the trainees interpreted 40 real studies. The results were promising. Some trainees performed as accurately as specialists (around 78% accuracy). Raters also showed reasonable agreement with each other. These results suggest that with even brief training, nonspecialists can learn to read FLIP Panometry studies reliably. They also suggest that FLIP could realistically be used widely and beyond just expert centers, though ongoing efforts to optimize training tools are still needed.

## Graphical abstract

Eight novice raters without motility experience completed brief FLIP panometry training and interpreted 40 studies using v1.0 and v2.0 classification schemes. Accuracy compared to expert consensus was high (v1.0: 0.78, v2.0: 0.73), with good‐to‐moderate interrater agreement (v1.0: κ 0.61, v2.0: κ 0.55).

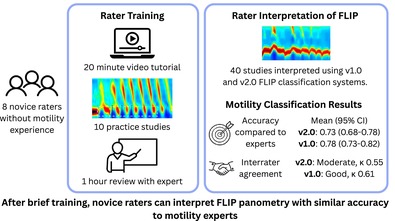

## 1 Introduction

Accurate diagnosis of esophageal motility disorders is essential to guide clinical management decisions. Functional lumen imaging probe (FLIP) Panometry, which utilizes impedance planimetry to measure esophageal luminal dimensions and esophageal distensibility in response to controlled volumetric distension during sedated endoscopy, has emerged as a useful tool for diagnosis of esophageal motility disorders [1, 2, 3]. FLIP Panometry evaluates the contractile response to esophageal distension (i.e., secondary peristalsis), as well as esophagogastric junction (EGJ) distensibility and opening, which provides esophageal motility assessments. FLIP Panometry assesses similar, but different aspects of esophageal function from high‐resolution manometry (HRM; which evaluates swallow‐associated primary peristalsis and deglutitive lower esophageal sphincter (LES) relaxation). Hence, FLIP with endoscopy can diagnose esophageal motility disorders, though in cases of “inconclusive” FLIP or HRM results, these can be used in a complementary manner with other clinical results (e.g., timed barium esophagram [TBE]) to determine a diagnosis [1, 2, 3, 4].

Real‐time interpretation of FLIP during endoscopy allows for efficient diagnosis for providers and patients [3]. Because FLIP is completed at the time of endoscopy (the typical initial evaluation among patients evaluated for esophageal symptoms such as dysphagia), it offers the potential to improve the efficiency for diagnosing esophageal motility disorders. Further, using FLIP with endoscopy can avoid some of the limitations with HRM that may lead to delayed diagnoses of motility disorders, such as inability to tolerate or place HRM catheters and delays associated with scheduling an additional encounter at an esophageal motility lab to complete HRM [3, 5, 6, 7, 8]. Thus, expanding accurate use of FLIP into general gastroenterology practices could provide significant benefits for identifying and diagnosing patients with esophageal motility disorders. However, adoption of FLIP outside specialized motility centers remains limited, likely reflecting a combination of factors including access to the technology and potential concern about implementing esophageal motility interpretation among endoscopists that do not routinely interpret motility studies.

Previous studies evaluating manometry demonstrated higher accuracy and interrater agreement with HRM over conventional line tracings in achalasia subtypes classification based on Chicago Classification and correlating HRM and FLIP findings [9, 10, 11, 12]. With FLIP Panometry, one previous study reported accuracy of mean (5–95th percentile) 78% (72%–81%) and moderate‐to‐good interrater agreement of FLIP Panometry motility diagnoses among 15 esophageal motility specialists (with varying levels of FLIP experience), with similar accuracy of 82% (78%–84%) for HRM [12]. Good‐to‐excellent inter and intrarater reliability of FLIP Panometry parameter measurements has also been described among 8 esophageal motility experts in healthy subjects [13]. However, studies assessing the ability of novice raters to interpret FLIP Panometry studies have not been completed, which has relevance for expansion and implementation of FLIP Panometry use to new users in clinical practice (particularly for non‐motility experts), as well as for motility curriculum for gastroenterology trainees. Further, the FLIP motility classification scheme was recently updated to reflect advances in FLIP Panometry motility evaluation: FLIP Panometry version 2.0 (the Dallas consensus); hence rater accuracy or reliability for interpretation remains unstudied with this new criteria [1, 2, 14].

This study aimed to assess the accuracy of novice raters' interpretation of FLIP Panometry studies after brief, simple training, using both the initial v1.0 and novel v2.0 FLIP Panometry motility classifications.

## 2 Methods

### 2.1 Subjects

**FIGURE 1:**
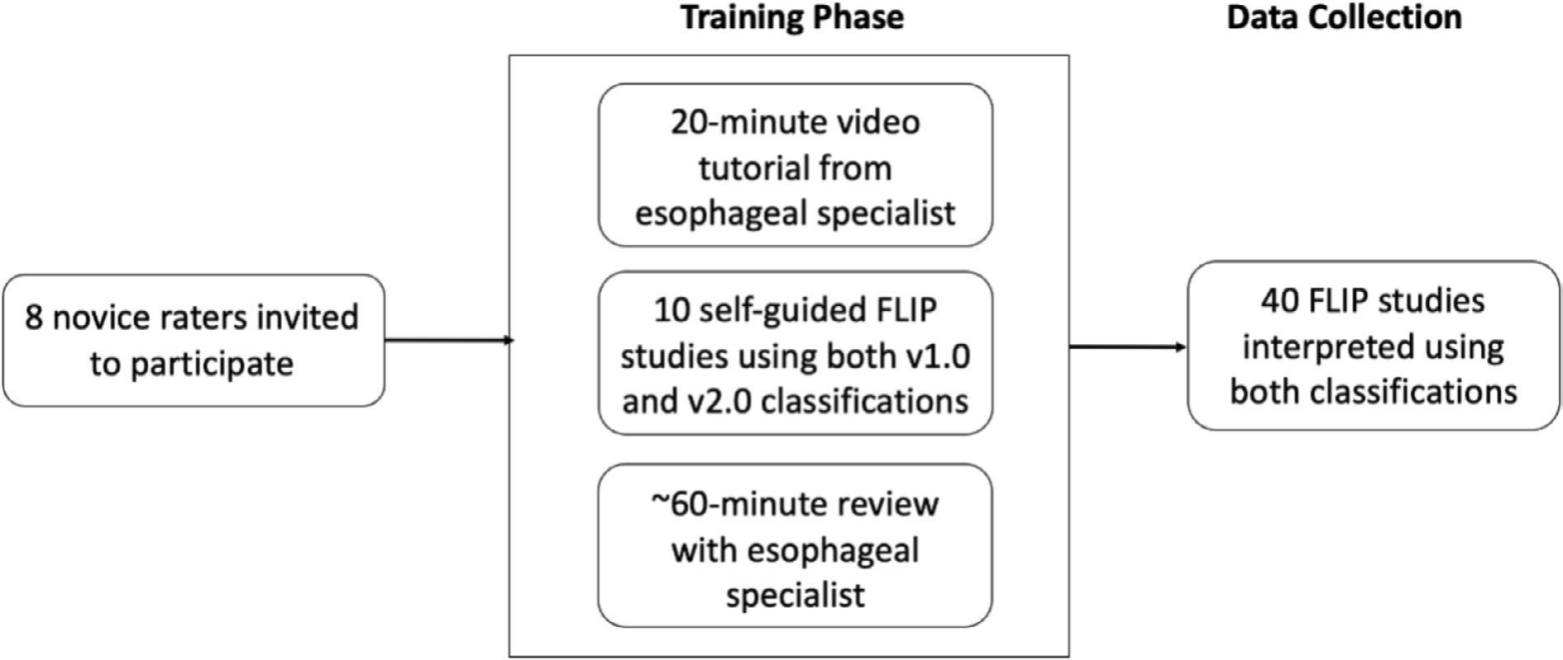
Study design overview for novice rater training and data collection. Eight novice raters underwent a structured training phase consisting of a 20‐min video tutorial, independent review of 10 functional lumen imaging probe (FLIP) studies using both v1.0 and v2.0 classification systems, and an approximately 60‐min case review session with an esophageal specialist. Following training, raters interpreted 40 FLIP studies using both classification systems during the data collection phase.

Eight raters (3 second‐year internal medicine residents, 4 first‐year internal medicine residents and 1 first‐year medical student) without previous FLIP or esophageal motility experience were recruited from an academic medical training center; Table 1. Novice raters also had not read the previously published study describing motility specialist interpretation of FLIP studies. All invited raters accepted the invitation to participate and completed the study; Figure 1.

**TABLE 1:** Rater characteristics.

	Raters; *n* (%)
Stage in training	
PGY2, Internal Medicine	3 (38)
PGY1, Internal Medicine	4 (50)
First‐year medical student	1 (13)
Gender	
Male	5 (63)
Female	3 (38)
Functional lumen imaging probe (FLIP) experience^a^	
0	8 (100)
≥ 1	0
High‐resolution manometry (HRM) experience^a^	
0	8 (100)
≥ 1	0
Upper endoscopy experience	
0	8 (100)
≥ 1	0

*Note:* Eight novice raters were included in this study. None of the participants had prior motility experience.

^a^ Reflects numbers of previous studies interpreted.

The deidentified FLIP studies were acquired from 40 consecutive adult patients evaluated for dysphagia at the Esophageal Center at Northwestern, who were prospectively enrolled in the parent study (P01 DK117824); Table S1. These were the same 40 patients analyzed in the previous study by esophageal motility specialists [12]. Patients with previous foregut surgery, mechanical obstruction or abnormal anatomy (e.g., strictures, hiatal hernia > 3 cm, or eosinophilic esophagitis) were excluded because these can be causes of secondary esophageal dysmotility. Raters were instructed that each patient was evaluated for dysphagia, had not had previous foregut surgery, and had completed upper endoscopy with no evidence of mechanical esophageal obstruction.

Informed consent was obtained from raters for study participation. The study protocol was approved by the Northwestern University Institutional Review Board.

### 2.2 Rater Training

Raters completed brief training that included a 20‐min video tutorial of FLIP v1.0 and v2.0 and access to the associated publications [1, 2, 15, 16]. The slides presented in the training video are included in File S1. Each rater then individually interpreted 10 practice FLIP Panometry studies. The practice studies (File S2) were selected to represent a spectrum of FLIP Panometry motility patterns and classifications. The practice cases were not included in the 40 cases used to measure rater accuracy. Each of the 10 practice studies were then reviewed over a 1‐h group meeting with an esophageal specialist (DAC), who described correct interpretations. The 10 training cases were interpreted via the html format output of the wklytics software (http://www.wklytics.com/nmgi); which was chosen given convenience and ease of creating, sharing, and using these plots.

### 2.3 FLIP Study Protocol and Analysis

The FLIP study using 16‐cm FLIP (EndoFLIP EF‐322 N; Medtronic) was performed during sedated endoscopy, as previously described [2, 12]. The FLIP study included stepwise 10‐mL FLIP distensions (each stepwise distension volume being maintained for 60 s) with the FLIP catheter positioned across the EGJ (1–3 intragastric channels).

Interpretation focused on the 50‐, 60‐, and 70‐mL fill volumes (based on the previously described FLIP classification schemes). The FLIP Panometry studies were reviewed by raters as deidentified recordings of real‐time FLIP Panometry studies (FLIP 2.0; Medtronic; these studies were completed prior to availability of the FLIP 300 system). Raters were instructed to view the recordings as they would during an endoscopic encounter, that is, continuous forward play of the recording with pause of the recordings allowed to review and/or record measures. Raters recorded values for EGJ distensibility index during 60 mL FLIP fill volume and maximum EGJ diameter (60–70 mL fill volume), classification of EGJ opening, classification of contractile response pattern (versions 1.0 and 2.0), and classification of esophageal motility (versions 1.0 and 2.0) into standardized data collection forms.

To assess accuracy, interpretations by the study participants were compared against a group consensus interpretation by two experienced raters (JEP and DAC) who created reference diagnoses for each FLIP study. Consensus on a reference diagnosis was able to be reached on 100% of studies.

### 2.4 Statistical Analysis

We computed confusion matrices of rater responses relative to reference diagnoses for all 320 responses (8 × 40) as well as by rater. Evaluation of trainee performance relative to reference diagnoses focused on accuracy and balanced accuracy, which were computed along with 95% confidence intervals overall and by rater. An accuracy of 78%, which was the mean accuracy for esophageal motility specialists relative to reference standard for FLIP Panometry v1.0 motility classification, was utilized as the primary benchmark for novice raters in this study, with 72% accuracy (5th percentile among esophageal motility specialists) was also utilized [12]. There were no studies with a FLIP motility classification of obstruction with normal contractility, hence this was omitted from confusion matrix tables. For continuous variables (EGJ‐DI, pressure at EGJ‐DI, and maximum EGJ diameter), we calculated the mean (standard deviation) difference between rater values and the reference values, as well as the standard error (SE). Interrater agreement was assessed using intraclass correlation coefficient (ICC) for continuous variables and Fleiss' kappa statistic for nominal variables. The degree of agreement for ICC and kappa values were defined as follows: slight agreement (0.00–0.20), fair (0.21–0.40), moderate (0.41–0.60), good for (0.61–0.80), and excellent (0.81–1.0) [17].

## 3 Results

### 3.1 Raters

Among the 8 raters that completed the study, none had previous experience of interpreting FLIP Panometry or HRM, nor did any have experience performing upper endoscopy Table 1. Raters reported time to complete interpretation of the 40 studies of mean 3.6 h (SD 1.1).

### 3.2 Accuracy of Interpretation of FLIP Panometry Motility Classifications

Accuracy of FLIP Panometry motility classification was mean 0.73 (95% CI 0.68–0.78; balanced accuracy = 0.72) for FLIP Panometry v2.0 and 0.78 (0.73–0.82; balanced accuracy = 0.77) for FLIP Panometry v1.0. There were 4 novice raters who had an accuracy ≥ 78% for both schemes and 5 raters with an accuracy > 72%. Accuracies ≥ 78% were achieved by three raters for v2.0 and five for v1.0 while accuracies of > 72% were achieved by five raters for v2.0 and six raters for v1.0; Table 2.

**TABLE 2:** Accuracy of esophagogastric junction opening classification, contractile response pattern, and motility diagnosis for Functional Lumen Imaging Probe (FLIP) v1.0 and FLIP 2.0 for each rater.

Accuracy by rater							
	EGJ opening	FLIP v1.0 CR	FLIP v1.0 motility	FLIP v2.0 CR	FLIP v2.0 motility	Average accuracy (overall)	Average accuracy (motility classifications)
Rater 1	0.88	0.88	0.83	0.93	0.88	0.88	0.85
Rater 2	0.88	0.78	0.88	0.83	0.88	0.85	0.88
Rater 3	0.90	0.83	0.88	0.78	0.83	0.84	0.85
Rater 4	0.88	0.63	0.78	0.63	0.73	0.73	0.75
Rater 5	0.68	0.80	0.73	0.85	0.63	0.74	0.68
Rater 6	0.70	0.73	0.70	0.75	0.63	0.70	0.66
Rater 7	0.83	0.85	0.85	0.78	0.75	0.81	0.80
Rater 8	0.65	0.80	0.60	0.75	0.55	0.67	0.58

*Note:* Eight novice raters interpreted 40 FLIP studies. Rater classifications for EGJ opening, contractile response pattern, and motility classification were compared to a consensus reference standard established by two motility specialists.

Abbreviations: CR, contractile response; EGJ, esophagogastric junction.

Using the v2.0 system, all 51 rater diagnoses of normal motility had a reference label as normal; Table 3. For the studies with a reference diagnosis of normal using v2.0, there were zero rater diagnoses of spastic obstruction or non‐spastic obstruction. Among the 86 inaccurate FLIP Panometry v2.0 motility diagnoses, “possible obstruction” was involved in 58 (67%); Table 3.

**TABLE 3:** Accuracy of functional lumen imaging probe (FLIP) panometry motility classifications for FLIP panometry version 2.0 classification scheme.

Accuracy of motility classifications using FLIP v2.0 classification						
Rater classification	Reference classification					
	Normal *n* (%)	Hypo‐contractility *n* (%)	Possible spasm *n* (%)	Possible obstruction *n* (%)	Spastic obstruction *n* (%)	Non‐spastic obstruction *n* (%)
Total	*9 (100)*	*5 (100)*	*3 (100)*	*11 (100)*	*5 (100)*	*7 (100)*
Normal	51 (71%)	0	0	0	0	0
Hypo‐contractility	1	35 (88%)	5	4	0	0
Possible Spasm	6	0	15 (63%)	2	0	0
Possible obstruction	14	4	4	60 (68%)	7	1
Spastic obstruction	0	0	0	10	18 (45%)	0
Non‐spastic obstruction	0	1	0	12	15	55 (98%)

*Note:* Eight novice raters each interpreted 40 FLIP studies using the v2.0 classification system. Rater classifications are shown in rows and reference (expert) classifications in columns. Shaded boxes represent accurate interpretations; percentages represent accurate interpretation for each reference motility classification by the novice raters. Non‐italicized values in table represent the pooled rater interpretations (8 interpretations per study). Italicized values indicate the number of studies per reference label in the 40‐study set.

Using v1.0, 51/52 (98%) rater diagnoses of normal motility had a reference label as normal; there was only one inaccurate rater diagnosis of normal motility that was labeled as “weak”; Table 4. For studies with a reference diagnosis of normal, there were zero rater labels of obstruction with weak CR. Among the 71 inaccurate FLIP Panometry v1.0 motility diagnoses, “inconclusive” was involved in 55 (71%); Table 4.

**TABLE 4:** Accuracy of Functional Lumen Imaging Probe (FLIP) Panometry motility classifications for FLIP Panometry version 1.0 classification scheme.

Accuracy of motility classifications using FLIP v1.0 classification					
Rater classification	Reference classification				
	Normal *n* (%)	Weak *n* (%)	Obstruction w/weak CR *n* (%)	Spastic reactive *n* (%)	Inconclusive *n* (%)
Total	*9 (100)*	*7 (100)*	*10 (100)*	*7 (100)*	*7 (100)*
Normal	51 (71%)	1	0	0	0
Weak	2	49 (88%)	0	1	4
Obstruction w/weak CR	0	0	74 (93%)	7	11
Spastic reactive	5	0	0	37 (66%)	3
Inconclusive	14	6	6	11	38 (68%)

*Note:* Eight novice raters each interpreted 40 FLIP studies using the v1.0 classification system. Rater classifications are shown in rows and reference (expert) classifications in columns. Shaded boxes represent accurate interpretations; percentages represent accurate interpretation for each reference motility classification by the novice raters. Non‐italicized values in table represent the pooled rater interpretations (8 interpretations per study). Italicized values indicate the number of studies per reference label in the 40‐study set. CR, contractile response.

### 3.3 Accuracy of Interpretation of FLIP Panometry EGJ Opening

The mean difference (SD; SE) for the raters compared with the reference standard was −0.64 (0.33; 0.16) mm^2^/mmHg for EGJ‐DI, 1.9 (3.0; 1.4) mmHg for pressure at EGJ‐DI, and −0.4 (1.1; 0.4) mm for maximum EGJ diameter. Raters exhibited an 80% accuracy (95% CI 0.75–0.84) of interpreting EGJ opening. There were zero rater labels of normal EGJ opening (NEO) among studies with a reference diagnosis of reduced esophageal opening (REO) and only one rater label of REO among studies with a reference label of NEO; Table 5. Hence, 64/65 (99%) of “inaccurate” esophageal opening interpretations involved the borderline/inconclusive (v1.0/v2.0) diagnosis.

**TABLE 5:** Accuracy of Functional Lumen Imaging Probe (FLIP) Esophagogastric Junction (EGJ) opening classifications.

Accuracy of EGJ opening classifications			
Rater classification	Reference classification		
	Normal *n* (%)	Borderline/Inconclusive *n* (%)	Reduced *n* (%)
Total	*16 (100)*	*12 (100)*	*12 (100)*
Normal	106 (83%)	13	0
Borderline/Inconclusive	21	60 (63%)	7
Reduced	1	23	89 (93%)

*Note:* Eight novice raters each interpreted the EGJ opening classification of 40 FLIP studies. Rater classifications are shown in rows and reference (expert) classifications in columns. Shaded boxes represent accurate interpretations; percentages represent accurate interpretation for each reference motility classification by the novice raters. Non‐italicized values in s represent the pooled rater interpretations (8 interpretations per study). Italicized values indicate the number of studies per reference label in the 40‐study set.

### 3.4 Accuracy of Interpretation of FLIP Panometry Contractile Response Patterns

For contractile response pattern, overall accuracy was mean 0.78 (95% CI 0.74–0.83) for FLIP v2.0 and 0.78 (0.74–0.83) for FLIP v1.0. For FLIP v2.0, the diminished or disordered contractile response patterns were involved in 89% of inaccuracies (57/69); Table 6.

**TABLE 6:** Accuracy of Functional Lumen Imaging Probe (FLIP) Panometry contractile response classifications for FLIP Panometry versions 2.0 and 1.0 classification schemes.

Accuracy of contractile response classifications using FLIP v2.0 classification						Accuracy of contractile response classifications using FLIP v1.0 classification					
Rater classification	Reference classification					Rater classification	Reference classification				
	Absent *n* (%)	Diminished *n* (%)	Normal *n* (%)	Disordered *n* (%)	Spastic *n* (%)		Absent *n* (%)	Borderline *n* (%)	Normal *n* (%)	Impaired/disordered *n* (%)	Spastic‐reactive *n* (%)
Total	*17 (100)*	*2 (100)*	*9 (100)*	*6 (100)*	*6 (100)*	Total	*18 (100)*	*1 (100)*	*8 (100)*	*6 (100)*	*7 (100)*
Absent	117 (86%)	2	0	12	3	Absent	119 (83%)	0	0	12	3
Diminished	16	8 (50%)	1	10	4	Borderline	1	6 (75%)	4	0	1
Normal	0	1	65 (90%)	0	1	Normal	0	1	54 (84%)	1	0
Disordered	0	5	1	25 (52%)	4	Impaired/disordered	21	1	1	35 (73%)	15
Spastic	3	0	5	1	36 (75%)	Spastic‐reactive	3	0	5	0	37 (66%)

*Note:* Eight novice raters each interpreted 40 FLIP studies and classified the contractile response using both v2.0 (left) and v1.0 (right) classification systems. Rater classifications are shown in rows and reference (expert) classifications in columns. Shaded boxes represent accurate interpretations; percentages represent accurate interpretation for each reference motility classification by the novice raters. Non‐italicized values in table represent the pooled rater interpretations (8 interpretations per study). Italicized values indicate the number of studies per reference label in the 40‐study set.

For FLIP v1.0, the impaired/disordered contractile response pattern was involved in 74% of inaccurate contractile response classifications (51/69); Table 6.

### 3.5 Interrater Reliability

For motility classification, agreement was good for FLIP v1.0 (kappa 0.61) and moderate for FLIP v2.0 (kappa 0.55); Table 7. Good interrater agreement was seen for EGJ‐DI (ICC [95% CI] 0.83 [0.75–0.89]) and maximum EGJ diameter (0.82 [0.75–0.89]). Fleiss' kappa indicated moderate agreement for EGJ classification (kappa 0.60) and for contractile response pattern using both FLIP v1.0 and v2.0 schemes (kappa 0.62 and kappa 0.60, respectively); Table 7.

**TABLE 7:** Interrater agreement in Functional Lumen Imaging Probe (FLIP) interpretation for version 1.0 and version 2.0 classification schemes.

Interrater agreement in FLIP interpretation	
	ICC (95% CI)
EGJ DI@60 mL	0.83 (0.75–0.89)
Max EGJ diameter	0.82 (0.75–0.89)
	*kappa*
EGJ opening classification	0.60
FLIP v1.0 contractile response	0.62
FLIP v1.0 motility classification	0.61
FLIP v2.0 contractile response	0.60
FLIP v2.0 motility classification	0.55

*Note:* Eight novice raters interpreted 40 FLIP studies. Interrater agreement was assessed using intraclass correlation coefficient (ICC) for continuous measures and Fleiss' kappa for categorical classifications. Higher values indicate greater agreement among raters. Distensibility index (DI) of the esophagogastric junction (EGJ) was measured during 60 mL FLIP fill volume.

Abbreviation: ICC, Intraclass correlation coefficient.

## 4 Discussion

The main finding of this study was that novice raters, that is, medical trainees without previous experience in esophageal motility interpretation, could interpret FLIP Panometry studies at a level of accuracy akin to esophageal motility specialists after brief training. 63% of the novice raters achieved the average accuracy observed among esophageal motility specialists using the FLIP Panometry motility classification v1.0, while 50% achieved a similar level using the novel FLIP Panometry v2.0 from the Dallas consensus [1, 12]. Further, among the entire cohort of eight novice raters, novice raters' interpretation of a normal study never occurred among studies with a reference label consistent with achalasia (spastic obstruction and non‐spastic obstruction in v2.0 or obstruction with weak CR in v1.0), nor did rater labels of a FLIP achalasia‐pattern occur among studies with a reference diagnosis of normal motility. Thus, when “inaccuracies” occurred, they were often to adjacent categories along the spectrum of FLIP Panometry motility findings that would likely have been without major clinical ramifications. These findings support that motility interpretation with FLIP Panometry can be easily learned, which supports its feasibility for broad implementation into general clinical practice.

Establishing accurate esophageal motility diagnoses is important to direct use of appropriate and necessary treatments. Hence, several previous studies have evaluated the accuracy and interrater agreement for interpretation of esophageal motility studies [9, 10, 11, 12, 13]. This present study represents the first to examine interpretation of FLIP Panometry among novice raters, as well as the first to assess multi‐rater interpretation using FLIP Panometry motility classification v2.0 (Dallas consensus) [1]. Our previous study, which utilized this same group of 40 patient FLIP studies interpreted by 15 esophageal motility specialists, was the first to evaluate accuracy and interrater agreement of FLIP Panometry motility diagnoses in symptomatic patients, and demonstrated a mean (95% CI) accuracy of 78% (72–81) for FLIP Panometry motility classifications using the v1.0 scheme (the study was completed prior to initiation of the Dallas consensus process) [12]. While it is not unexpected that esophageal motility specialists appeared to have better accuracy than novice raters, it was notable that five of eight novice raters in this study achieved the average accuracy of specialists (78%) and six of eight achieving accuracy greater than the 5th percentile of esophageal motility specialist, using the FLIP Panometry motility v1.0 scheme after only brief training. Fewer novice raters (50%) appeared to achieve the average specialist‐equivalent threshold with the FLIP Panometry motility classification v2.0 (noting that specialist interpretation of v2.0 has not been measured), with also slightly lower agreement (kappa 0.48 with v2.0 vs. kappa 0.61 with v1.0). The minor difference in performance between v2.0 and v1.0 is likely related to the additional class of contractile response patterns and motility classification (i.e., more potential for error) within the v2.0 scheme. Overall, however, these results suggest that FLIP Panometry interpretation can be easily learned, even among medical trainees without motility (or dedicated gastroenterology) training. Accordingly, this suggests that gastroenterology trainees and general gastroenterology providers would be able to learn FLIP Panometry interpretation with similar ease, if not with improved performance given additional experience and training in diagnosing gastrointestinal disorders beyond the novice raters in this study. This would facilitate implementation of FLIP Panometry in a variety of clinical settings, for example, community and academic centers.

While this was the first study to assess novice raters' interpretation of FLIP Panometry interpretation, medical trainee (not necessarily trainees without prior GI or motility experience) interpretation of esophageal motility with HRM using manometry was assessed in several previous studies. A study of 44 gastroenterology fellows demonstrated that completion of a standardized HRM training program (on which trainees spent a mean (SD) 11.9 (8.6) hours) had post‐training diagnostic accuracy ranging from mean (SD) 63% (27%) for spastic disorders to 80% (27%) for normal motility, with 63% of fellows achieving “competency” [18]. Another study that included 36 medical trainees (students, residents, and fellows) that completed a 30‐min tutorial on HRM and line tracing manometry achieved a pooled accuracy for esophageal motility diagnosis of 57%–60.6% [11]. Although HRM interpretation was not incorporated into the present study and the prior studies' training methods differ from those of our study, the degree of accuracy with FLIP Panometry interpretation (even within our truly esophageal motility novice cohort) appeared to be equivalent, if not superior, to non‐esophageal motility specialists (i.e., trainees with varying degrees of gastroenterology and/or motility experience) interpreting HRM. The accuracy of novices in our study despite the relatively reduced training time compared to the previous (11.9 h) is also noted. Overall, these findings align with prior studies that show variability to achieve proficiency for motility interpretation among learners. There currently are no specific benchmarks for competency in esophageal motility interpretation (FLIP or HRM), hence ongoing development of training and competency assessment programs remains a work in progress.

Also notable in this study was that when diagnostic “inaccuracy” occurred among the novice raters, the inaccurate interpretations were often limited to inconclusive or borderline cases (which was similar to the prior study of FLIP Panometry interpretation among specialists), which in clinical practice would typically prompt supplemental or alternate confirmatory testing [12]. The complementary role of FLIP Panometry with high‐resolution manometry (HRM) among inconclusive cases (from FLIP or HRM) has been previously discussed [4, 12, 19]. Further, there were zero cases with an “achalasia‐like” pattern on FLIP that were called normal by the novice raters. A key statement of the Dallas consensus was that a major motor disorder is unlikely with a normal FLIP Panometry classification, which was supported by its modified Delphi process and independent literature review and summary [1, 20]. Hence, accurate identification of normal motility during endoscopy using FLIP Panometry may allow patients to avoid unnecessary additional testing (including discomfort associated with HRM), and avoid treatment delay [3, 12, 21]. The accuracy of novices in assigning normal motility further boosts support for using FLIP for this purpose.

While the rates of accuracy observed in the present study suggest promise to implement use of FLIP Panometry among non‐esophageal motility specialists, it remains possible that novel and emerging tools for FLIP Panometry interpretation could provide even better interpretation in real‐world practice. This (and previous) study utilized recorded videos from the FLIP 2.0 system, which can be paused to obtain measures during real‐time use but otherwise provides minimal interpretation output. The more recently available FLIP 300 system (Medtronic Inc) allows for studies to be paused and scrolled through (forward and reverse) in real‐time during endoscopy, which may improve interpretation. Further, post‐endoscopy analysis can be performed with archived or saved FLIP files using open‐source research software as previously described via software available at www.wklytics.com/nmgi or a novel Mechview platform (https://mechview.wklytics.com/analysis) [2, 22]. The MechView platform facilitates interpretation by filtering out dry catheter and movement artifact, as well as provides hierarchical decision support, to guide assignment of motility classifications per the Dallas consensus [1]. Further, artificial intelligence platforms were developed and are being refined, which are also expected to serve a valuable role in interpretation assistance and clinical decision support in the years ahead [23].

This study has several notable strengths, including enrollment of a truly GI‐motility naïve cohort of raters and use of the same 40 FLIP studies of consecutive esophageal motility patients as previous work [12], which facilitated comparison between esophageal motility experts and novices. However, the study also had limitations, including those similar to those addressed in the prior study of esophageal specialists [12]. A limitation was that the cohort of patient cases was from an esophageal referral center with a high rate of patients with achalasia, noting however that the use of consecutive patients meeting inclusion simulated the real‐life scenario of the center (as opposed to selecting specific cases), thus some non‐classic and borderline cases were included. While this patient cohort may somewhat limit generalizability to other practices, the cases did provide a sufficient sample size of achalasia, which is the most actionable diagnosis in esophageal motility. Our rater training process, which involved a one‐hour meeting with an esophageal specialist, may also limit generalizability, though also suggests a reasonable potential training plan to utilize for new FLIP users. Further, training and reference labels being performed by the same specialist may impose some degree of contamination, though a similar approach was used in studies of learners on manometry [11, 18]. HRM interpretation was not assessed among this group of novice raters; while this could have provided a direct comparison for FLIP Panometry interpretation for this cohort, trainee HRM interpretation has been assessed in multiple other studies and hence wasn't included here. Further, unlike other studies on trainees, this study was not designed to assess baseline abilities or observe learning curves for FLIP Panometry interpretations [18, 24]. Finally, this study focused on interpretation of the FLIP Panometry tracings; however, this may not completely translate to real‐world intra‐procedural FLIP interpretation during which other components, such as adequate FLIP positioning, are also involved.

In conclusion, the majority of novice raters demonstrated accuracy akin to esophageal motility specialists compared to reference diagnoses established by experienced raters when using FLIP v2.0 and FLIP v1.0 classification systems. While some novice raters were able to achieve interpretation accuracy to similar levels of esophageal motility specialists, there was variability in accuracy among learners which underscores the need for comprehensive and adaptable or personalized training methods for esophageal motility interpretation. Ongoing work remains necessary for development of competency benchmarks for esophageal motility interpretation with both FLIP Panometry and HRM. While ongoing refinement of both the FLIP Panometry diagnostic approaches and training platforms is anticipated, this study supports that FLIP Panometry interpretation can be readily learned, even by gastroenterology novices (i.e., beyond esophageal motility novices), which lends support for accurate and reliable utilization of FLIP Panometry in general gastroenterology practices, including among non‐specialist providers.

Table S1 provides demographic and clinical characteristics of the 40 patient cases included in the study. File S1 contains the slides from the video tutorial used for training novice raters on FLIP Panometry interpretation. File S2 includes the 10 practice cases and answer key used during the training protocol.

## Author Contributions

C.E.B. and A.J.K. contributed to drafting of the manuscript, data analysis (including participating as raters), data interpretation, and approval of the final version. J.M.S. contributed to data analysis, data interpretation, and approval of the final version. K.C., B.M.M., C.O., A.P., A.S., and M.A.S. contributed to data analysis (raters), editing of the manuscript, and approval of the final version. R.Y. contributed to data interpretation, editing the manuscript critically, and approval of the final version. J.E.P. contributed to obtaining funding, data interpretation, editing the manuscript critically, and approval of the final version. D.A.C. contributed to study concept and design, obtaining funding, drafting of the manuscript, data analysis, data interpretation, and approval of the final version.

## Funding

This work was supported by the National Institute of Diabetes and Digestive and Kidney Diseases, R01 DK137775, P01 DK117824.

## Conflicts of Interest

The authors declare no conflicts of interest.

## Disclosure

Rena Yadlapati: Consultant for Phathom Pharmaceuticals, StatLinkMD, Braintree Pharmaceuticals, Reckitt Benckiser Healthcare Ltd., Medtronic; Advisory Board: RJS Mediagnostix. John E. Pandolfino: Diversatek (Grant), Phathom Pharmaceuticals (Consulting); EndoGastric Solutions (Speaking; Consulting); Ethicon (Speaking; Consulting); Medtronic (Speaking, Consulting, Patent, License); Laborie (Consulting; License); Sandhill Scientific/Diversatek (Consulting, Speaking, Grant); Torax (Speaking, Consulting). Dustin A. Carlson: Medtronic (Speaking, Consulting, License); Diversatek (Consulting), Phathom Pharmaceuticals (Speaking, Advisory Board); Braintree (Consulting); Medpace (Consulting); Regeneron/Sanofi (Speaking, Consulting); Laborie (Consulting, License).

## Supporting information


**File S1:** Slides in FLIP interpretation video tutorial. The brief training of novice raters included a 20‐min instructional tutorial with the following slides. The tutorial covered the concept of FLIP panometry, interpretation using the v1.0 and v2.0 classification schemes, and key differences between the schemes.

**File S2:** Practice cases used for FLIP interpretation training. Following the video tutorial, novice raters interpreted 10 practice studies and then reviewed correct interpretations with an esophageal specialist. The 10 cases were selected to represent a spectrum of FLIP panometry motility patterns and classifications.

**Table S1:** Case (patient) demographics and characteristics. Raters interpreted FLIP studies of 40 patients evaluated for dysphagia at the Esophageal Center of Northwestern. Patients with previous foregut surgery, mechanical obstruction, or abnormal anatomy were excluded from the study due to risk of secondary esophageal dysmotility. Raters were informed that all patients presented with dysphagia, had no prior foregut surgery, and had no evidence of mechanical obstruction on upper endoscopy.

## Data Availability

The data that support the findings of this study are available from the corresponding author upon reasonable request.
