# Supplementary material for: Accuracy of Novice Raters for Esophageal Motility Classifications Using Functional Lumen Imaging Probe Panometry
Source: Neurogastroenterol Motil. 2026 Jul 5;38(7):e70386. doi: 10.1111/nmo.70386 (PMC13334194; doi:10.1111/nmo.70386)

Supplementary File S1. Slides in FLIP interpretation video tutorial. The brief training of novice raters included a 20-minute instructional tutorial with the following slides. The tutorial covered the concept of FLIP panometry, interpretation using the v1.0 and v2.0 classification schemes, and key differences between the schemes.


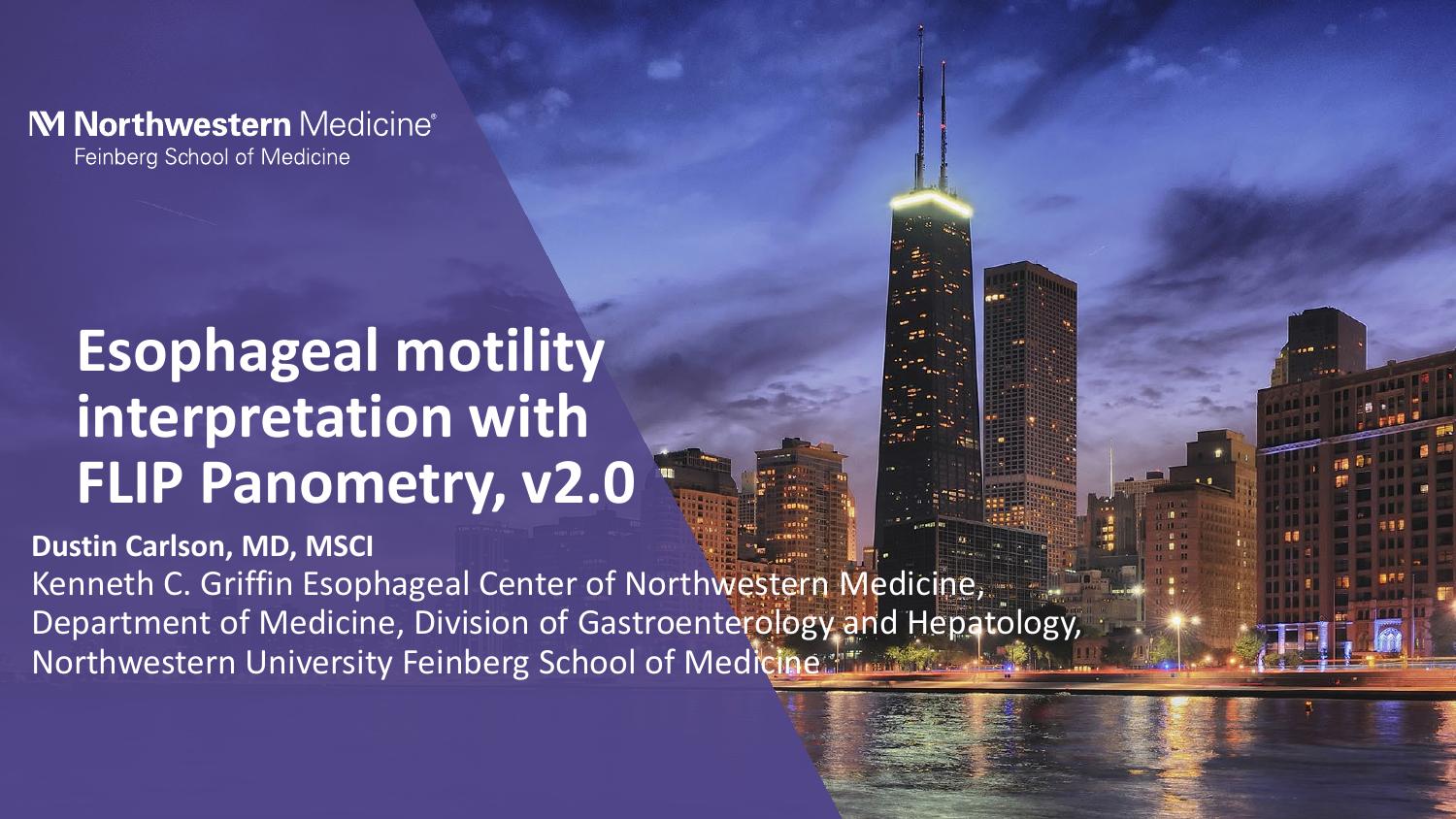


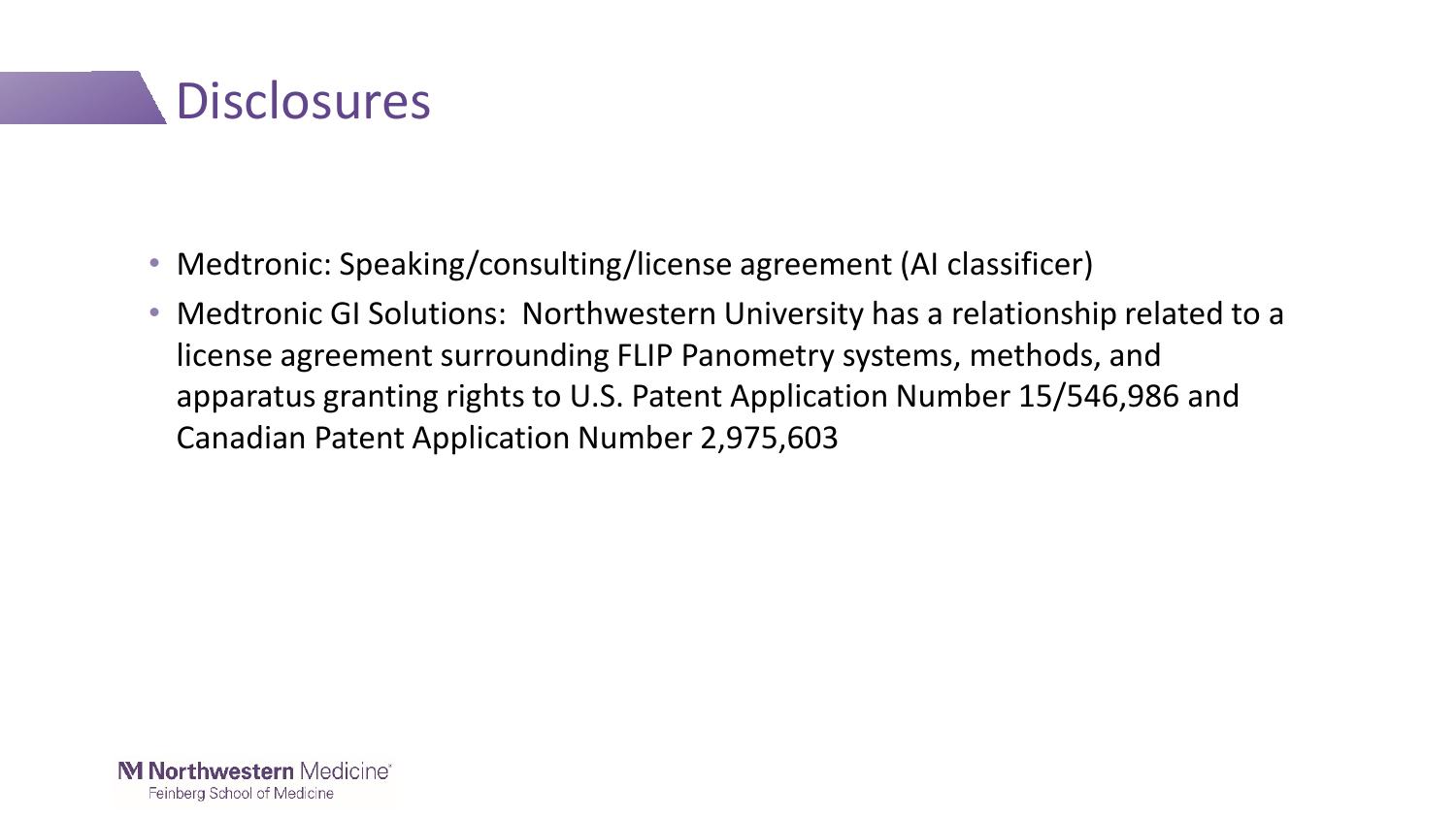


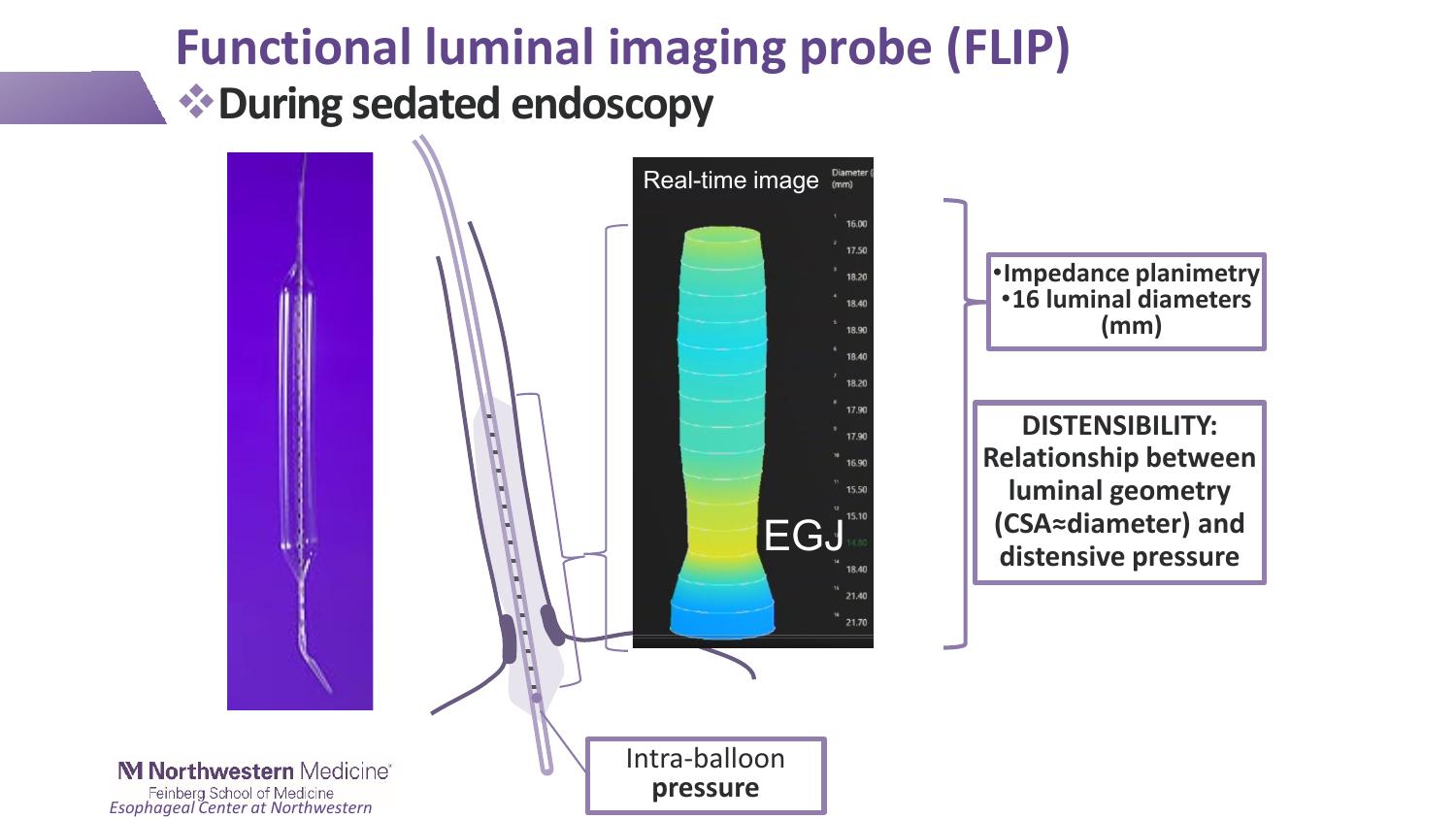


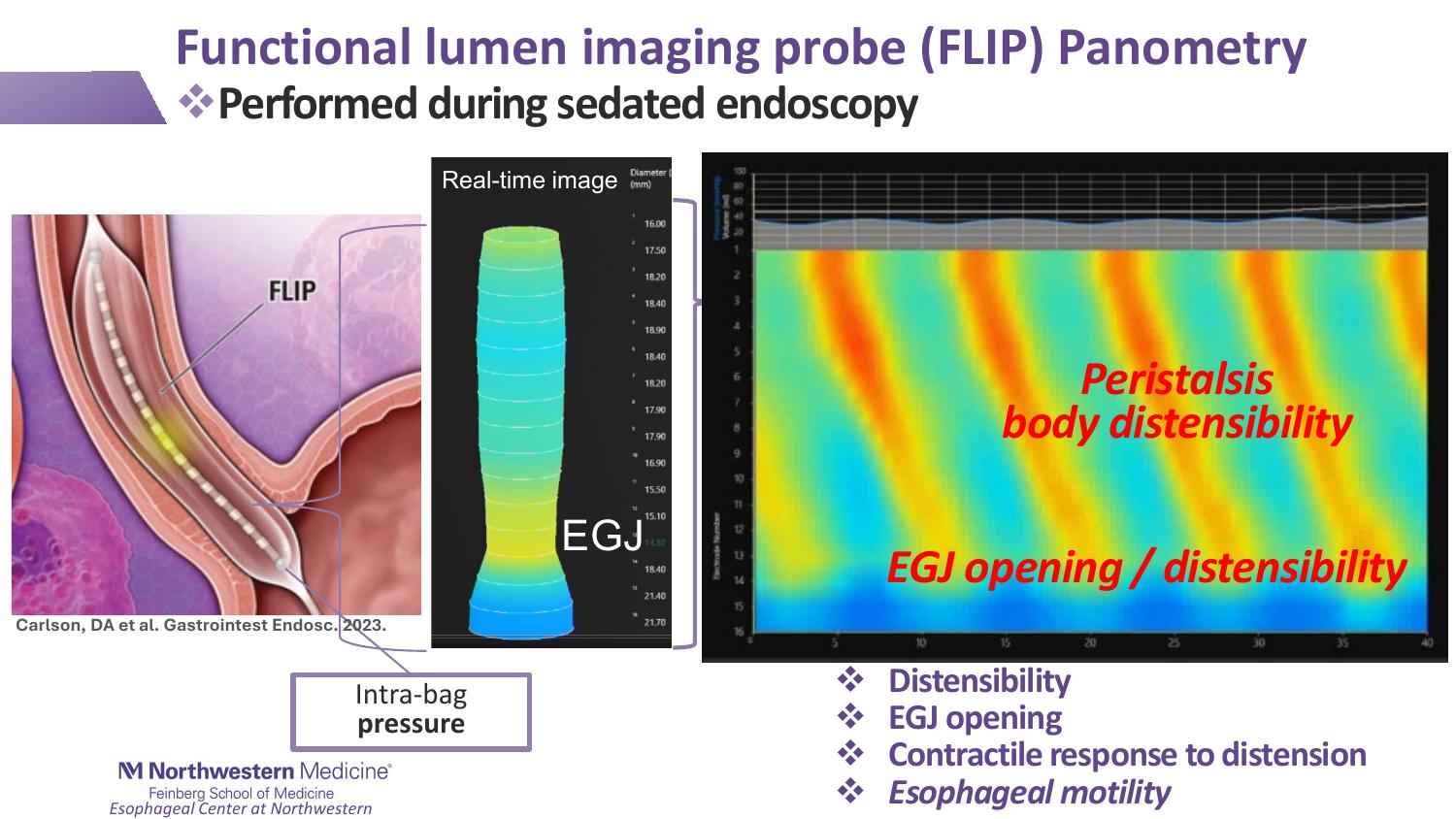


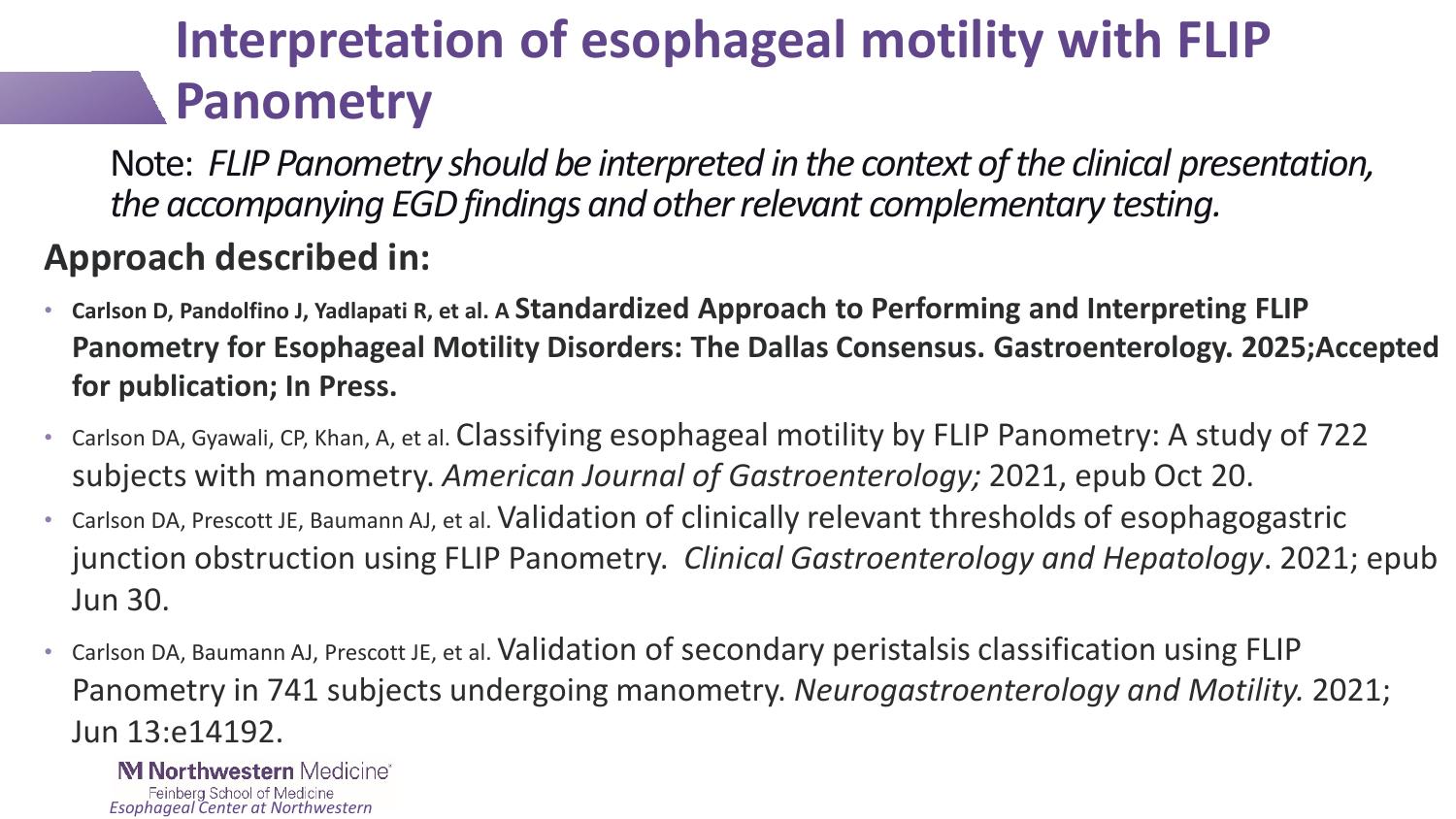


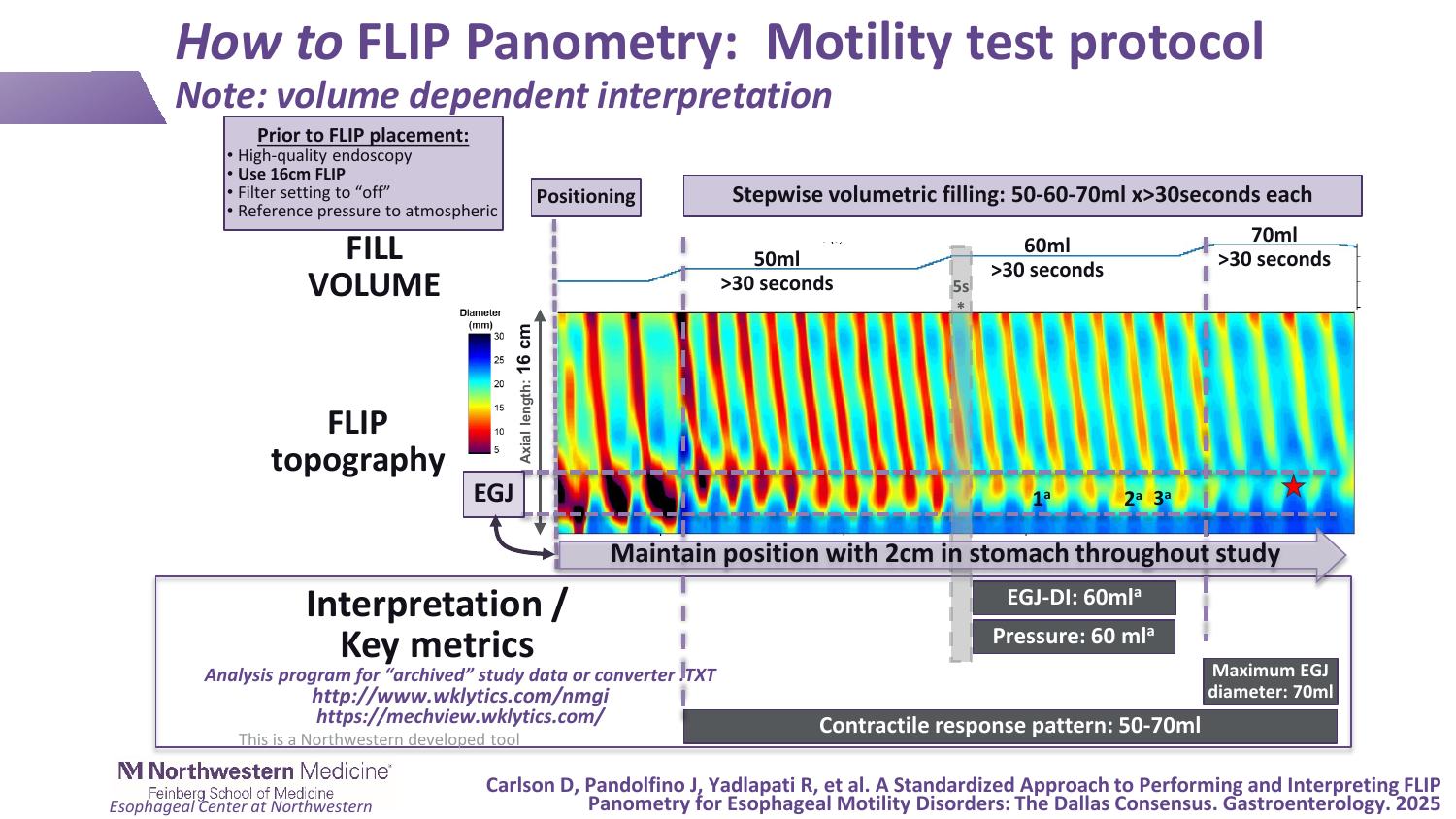


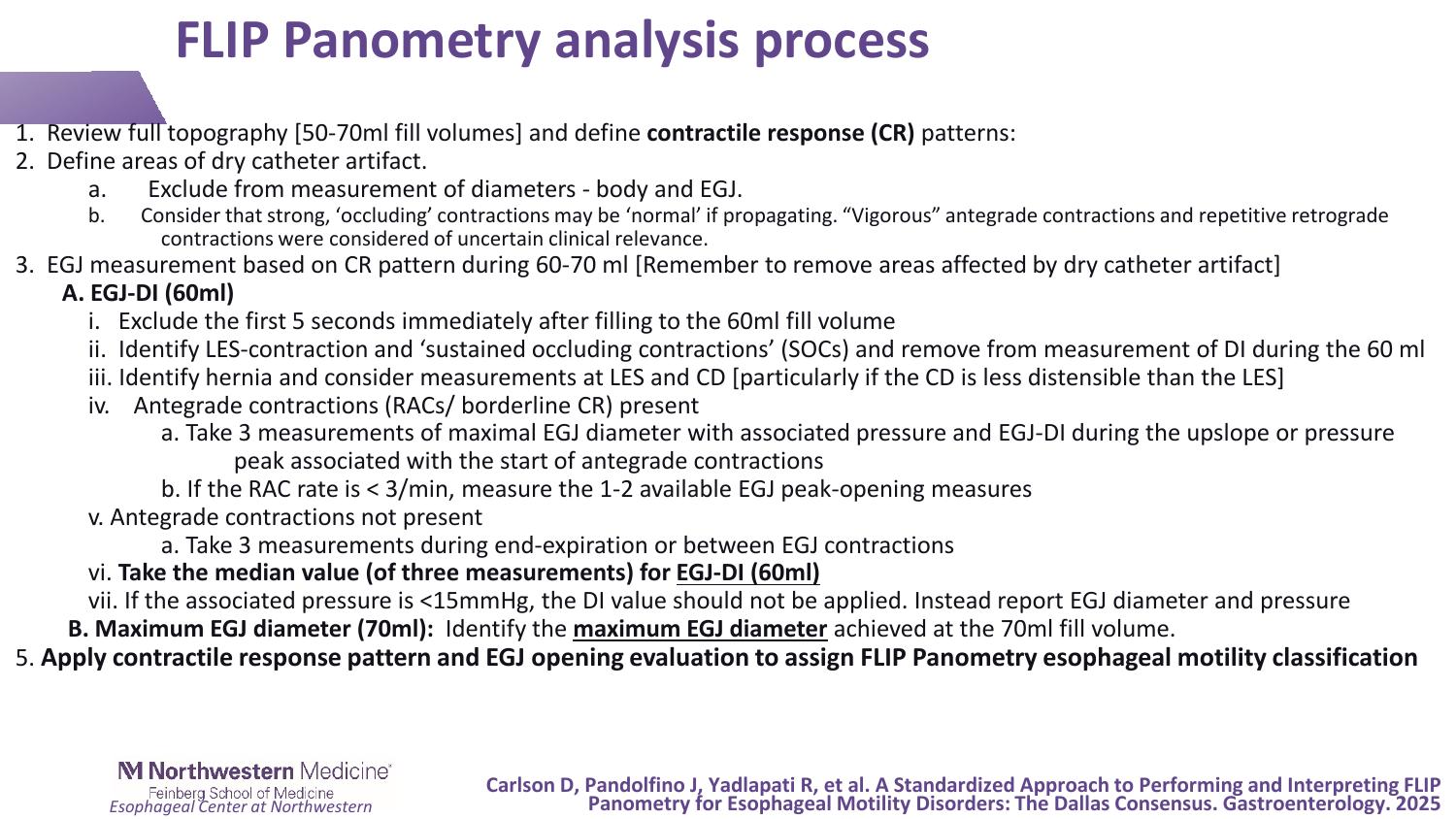


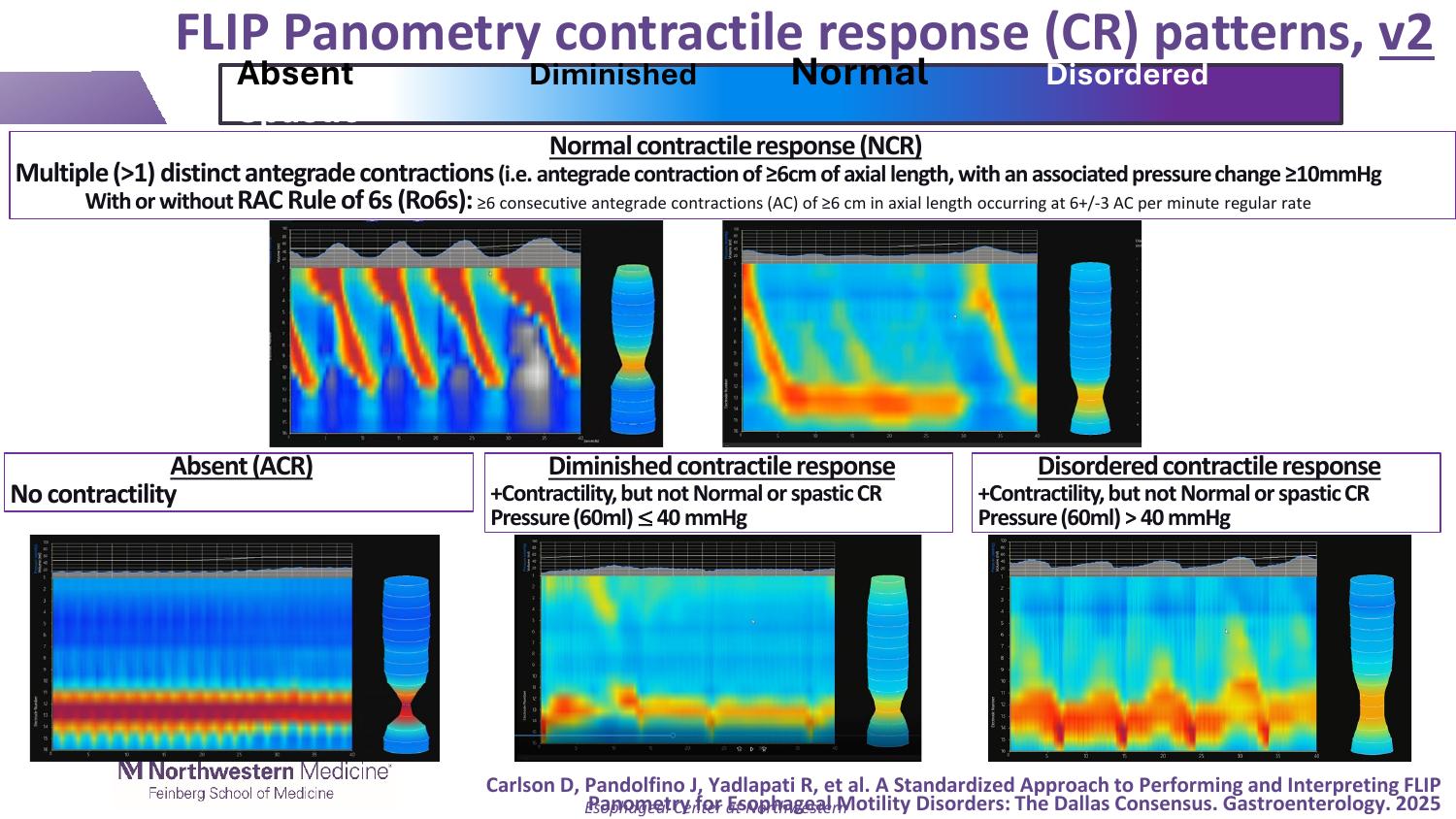


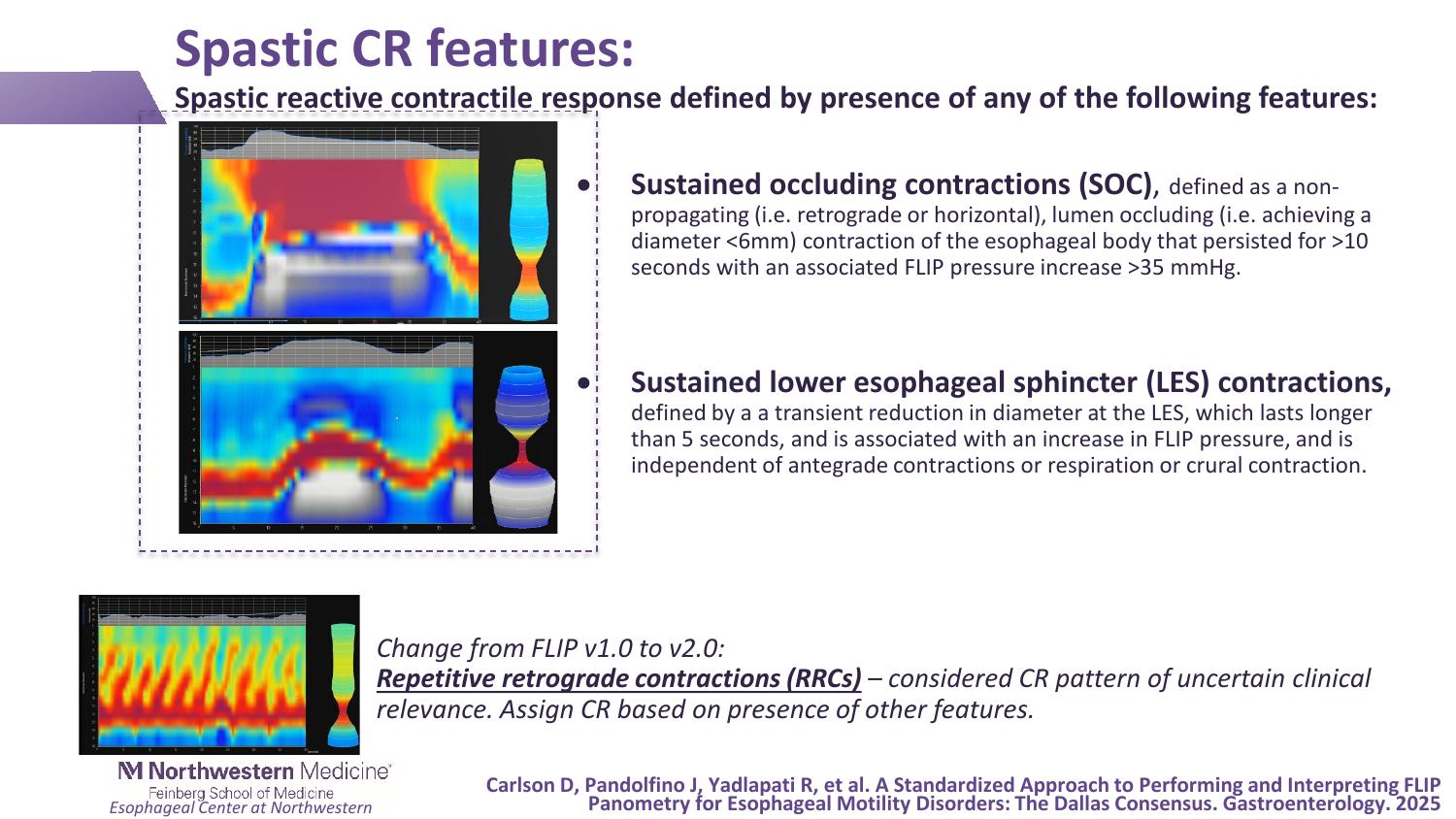


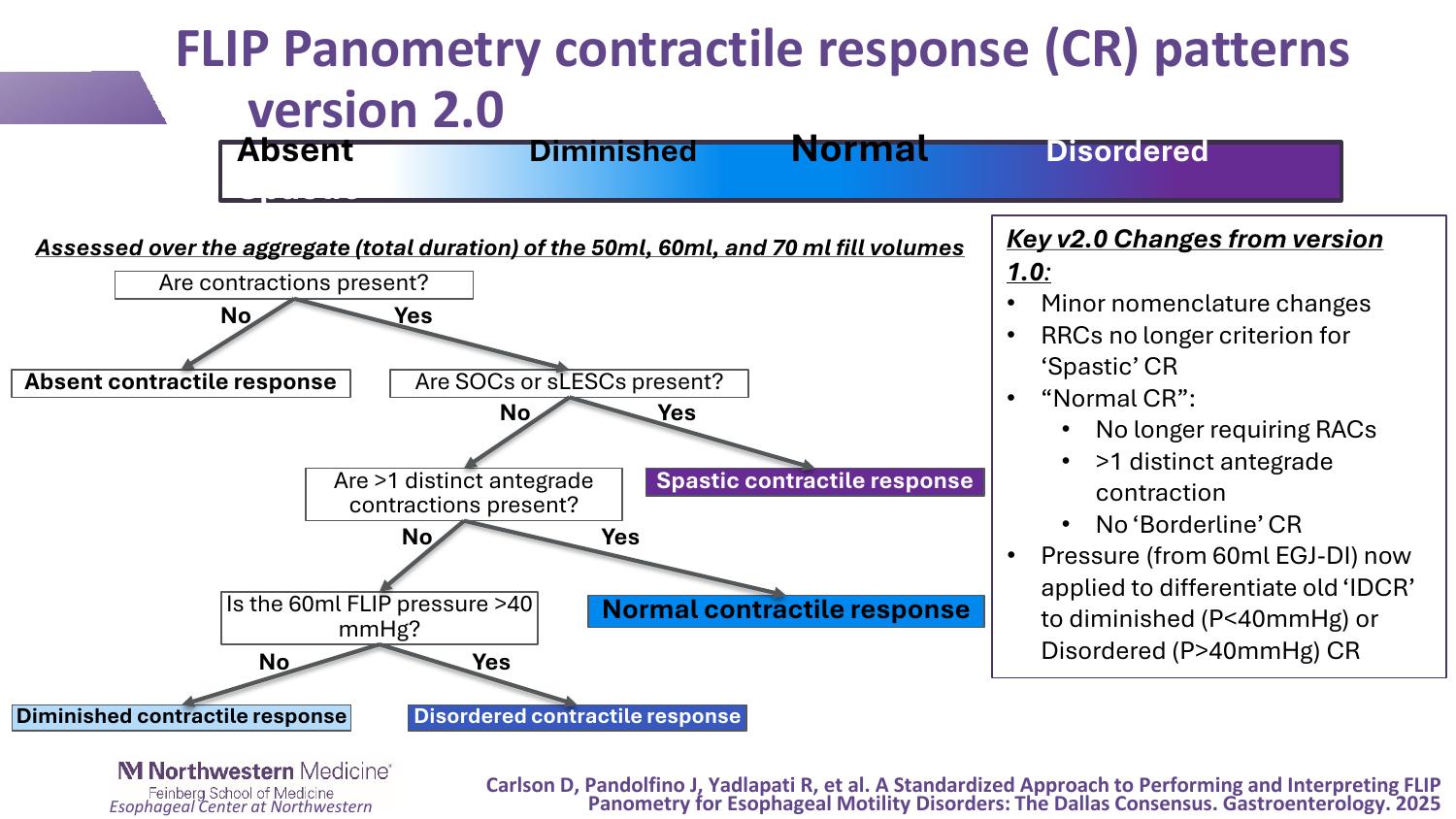


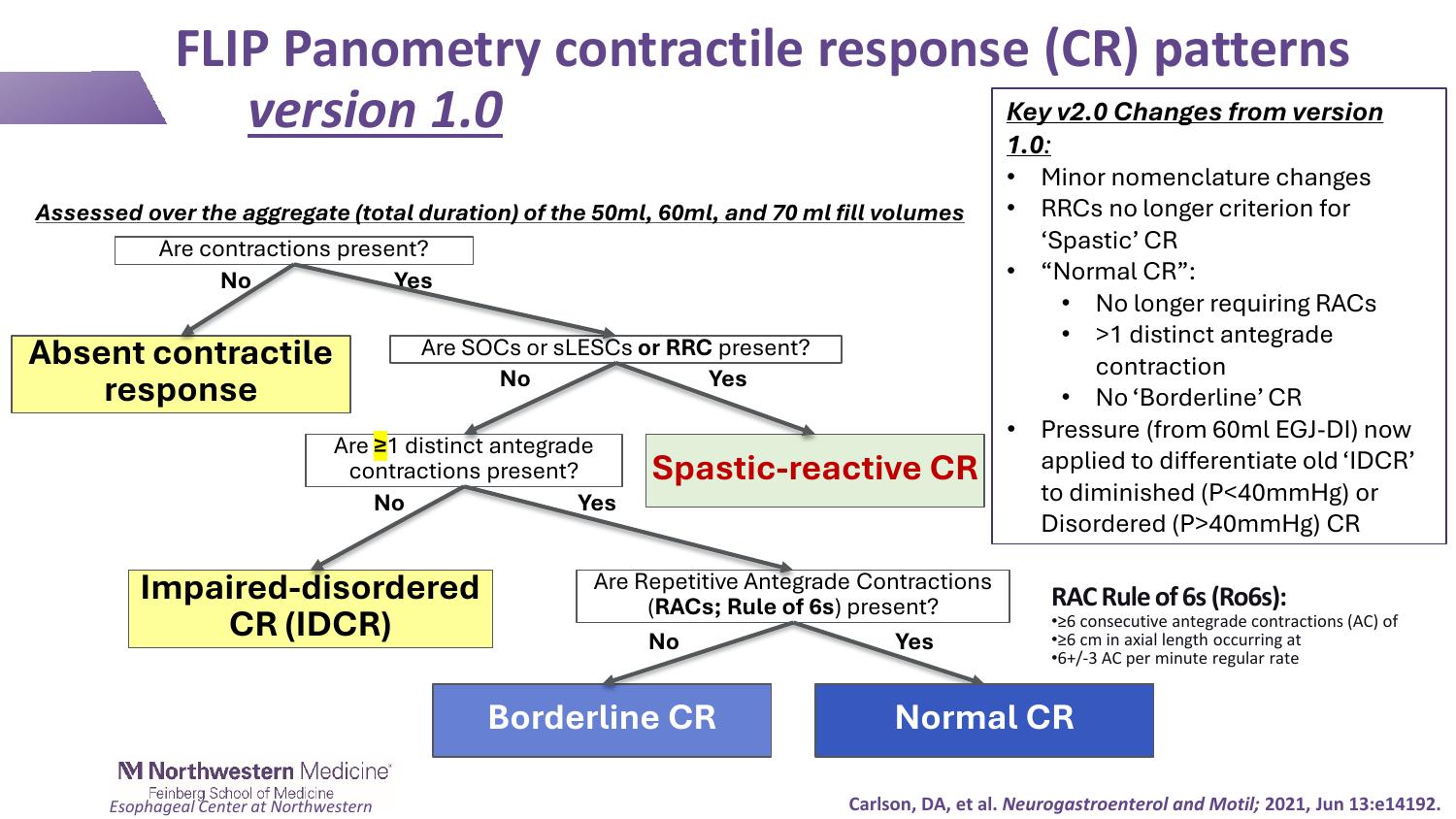


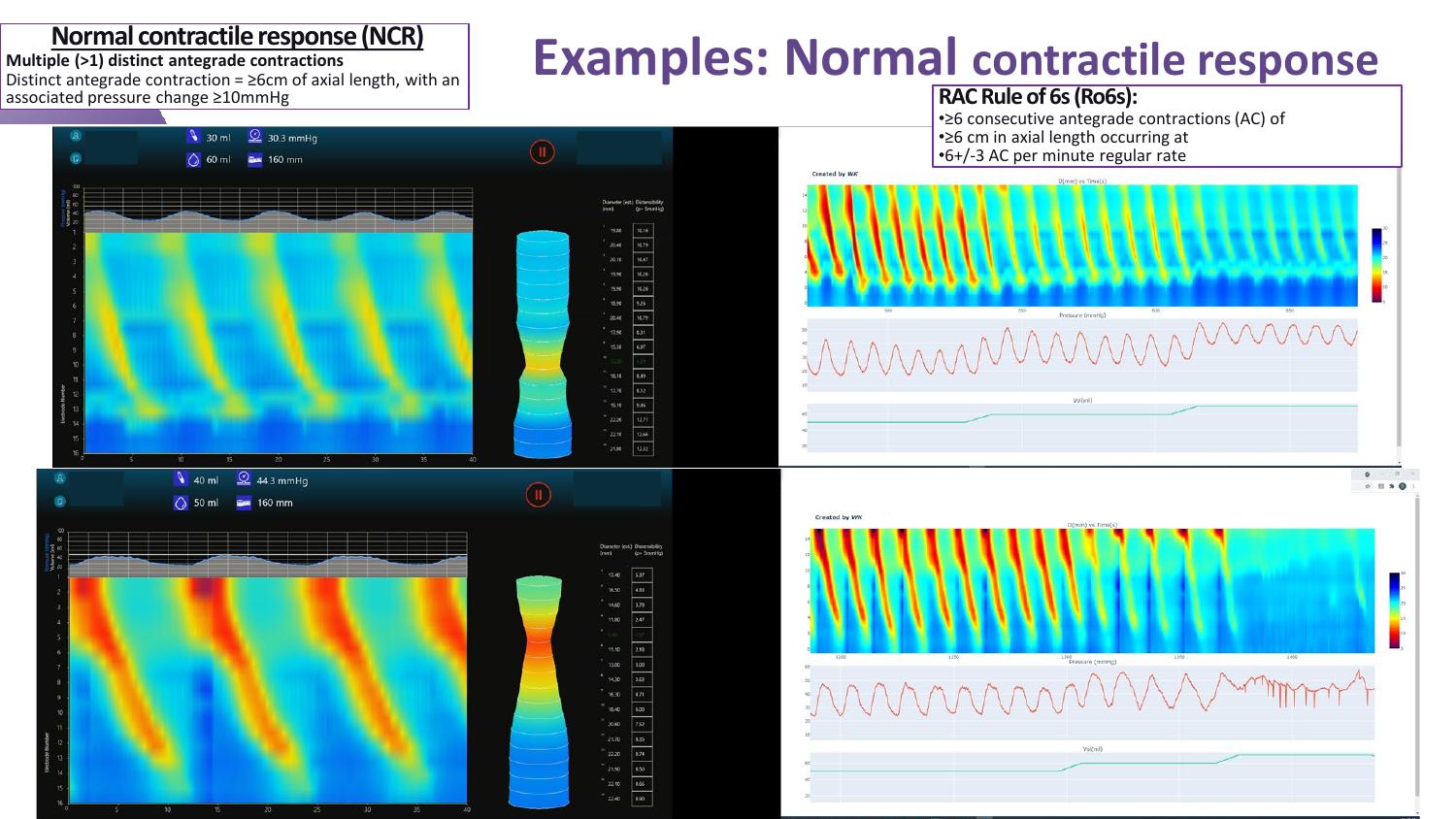


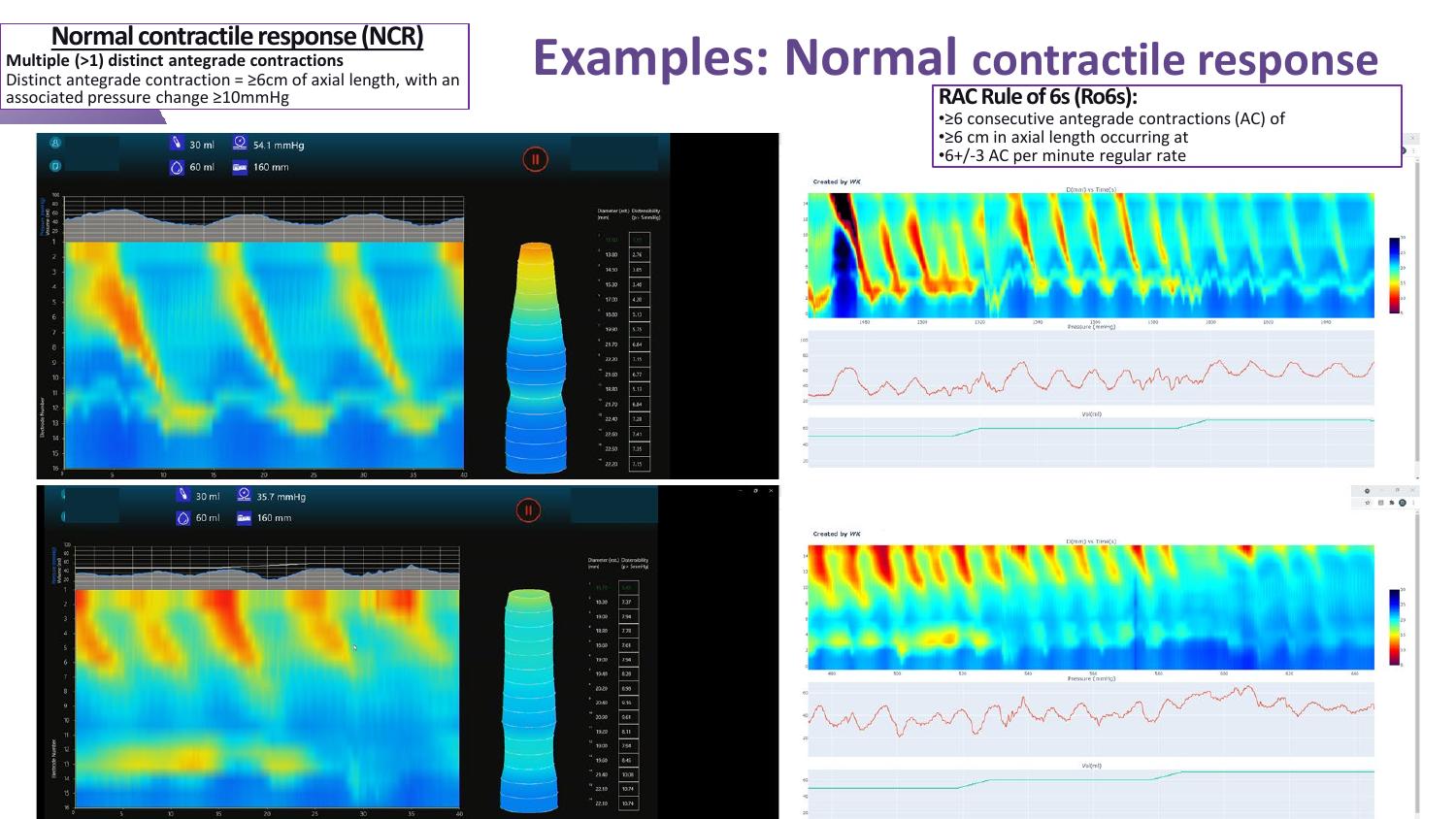


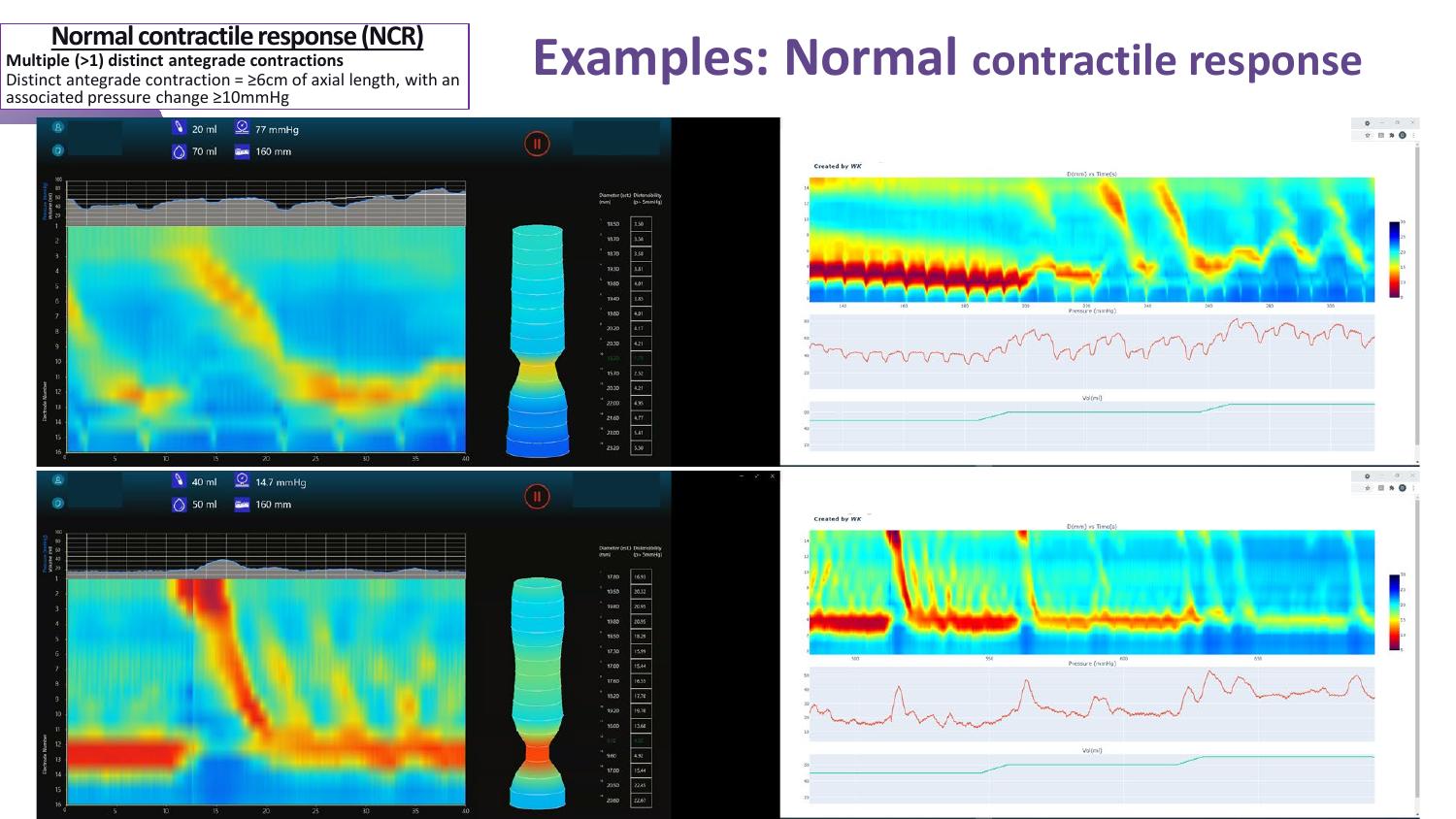


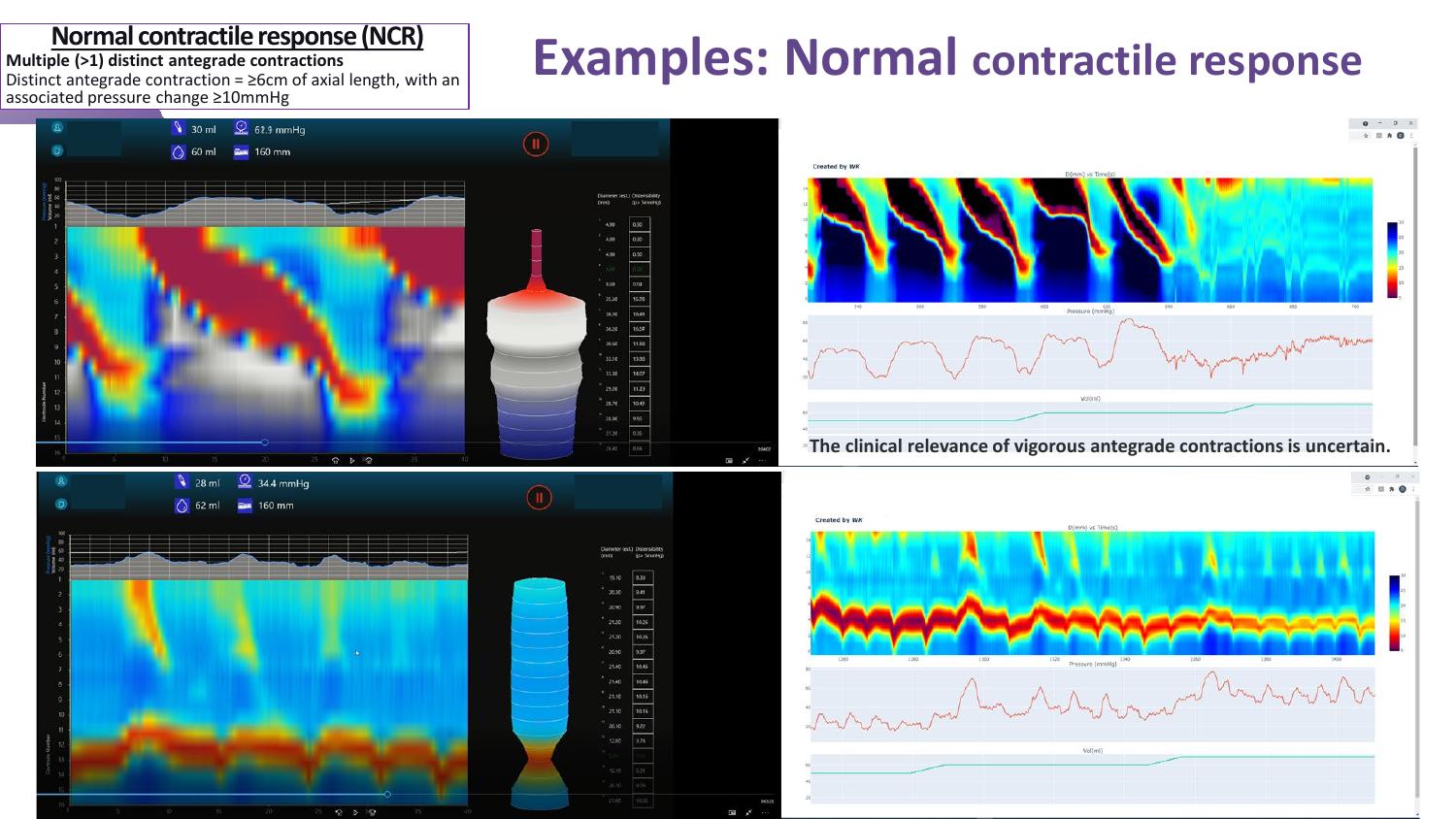


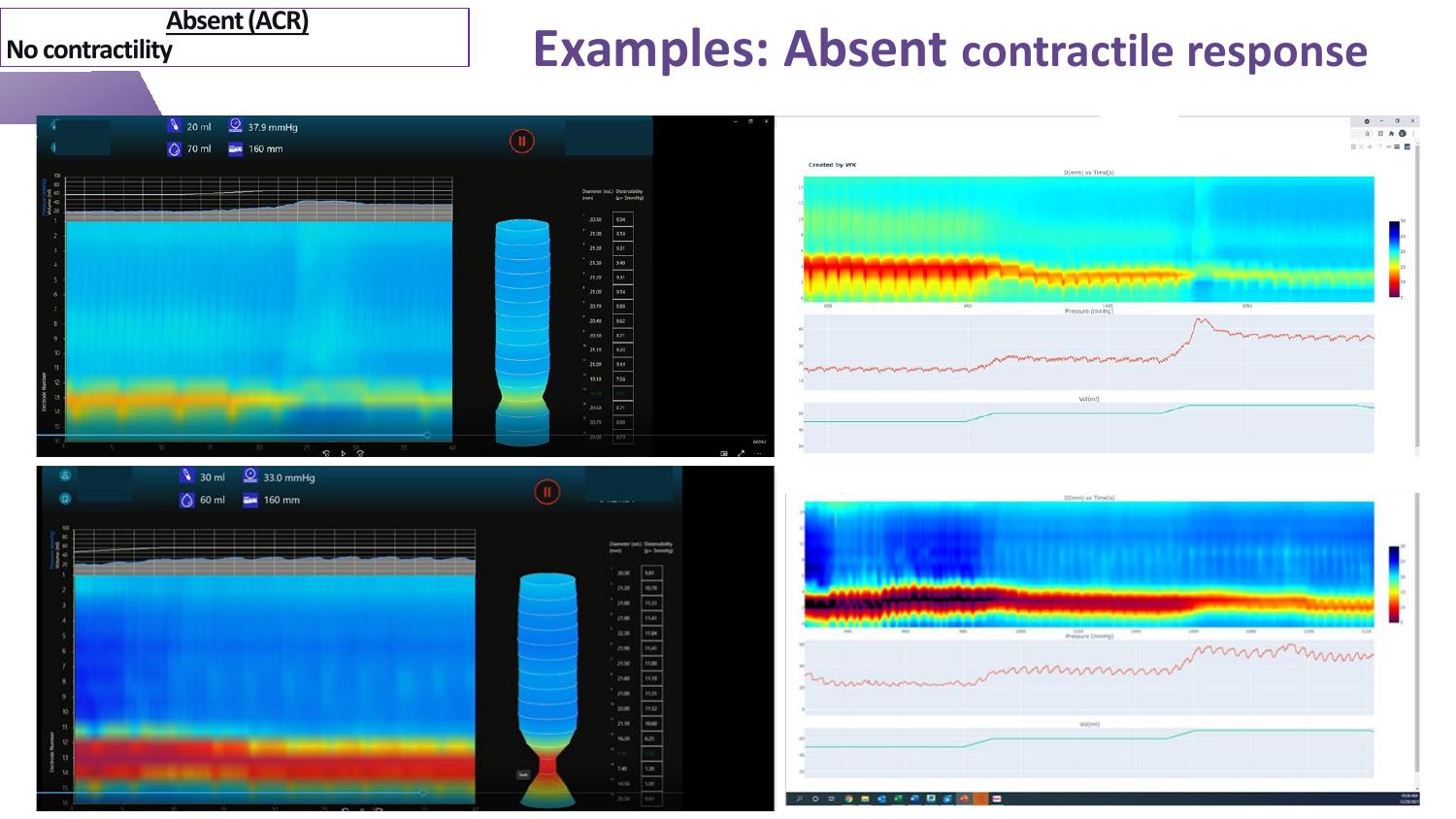


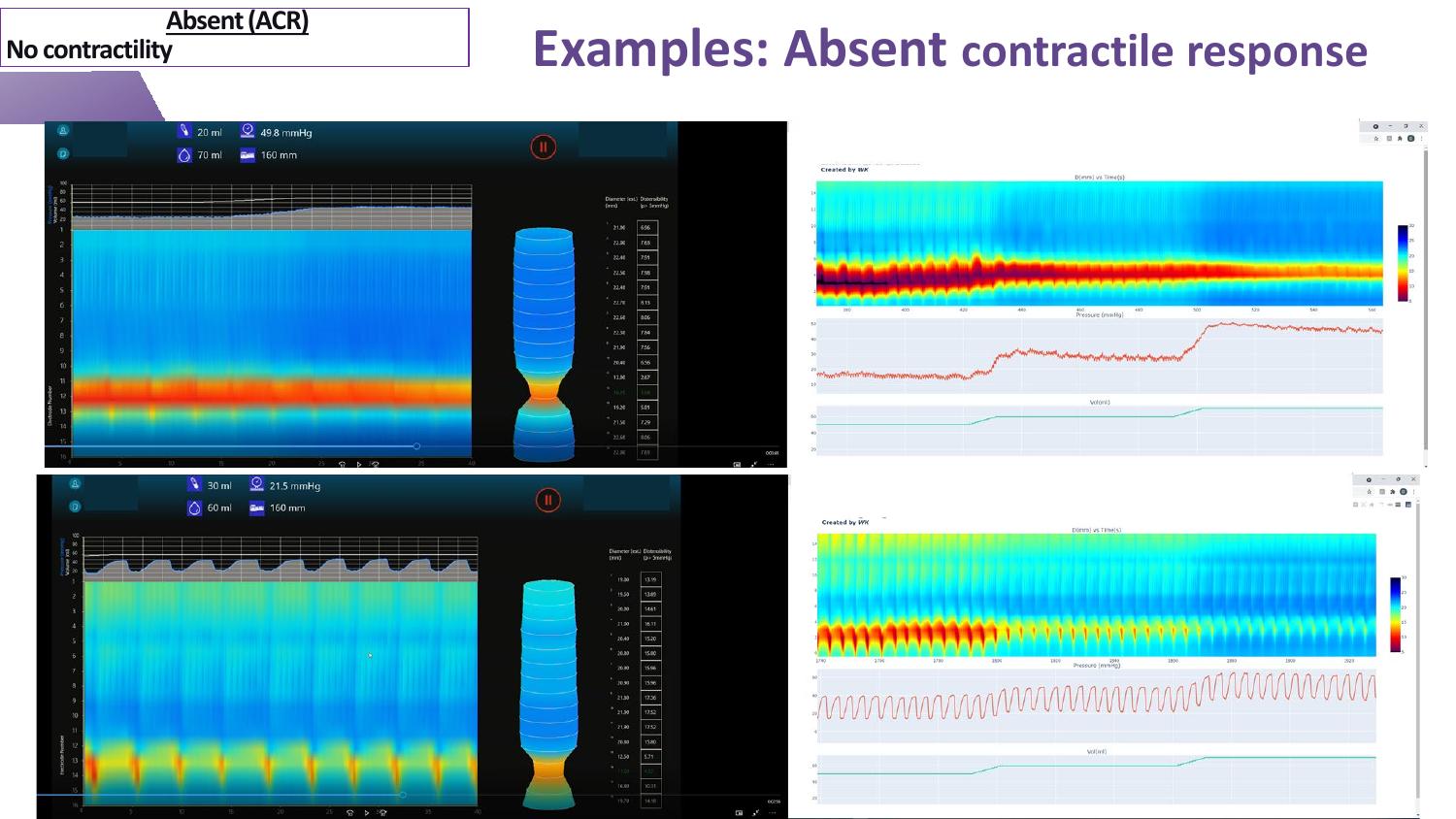


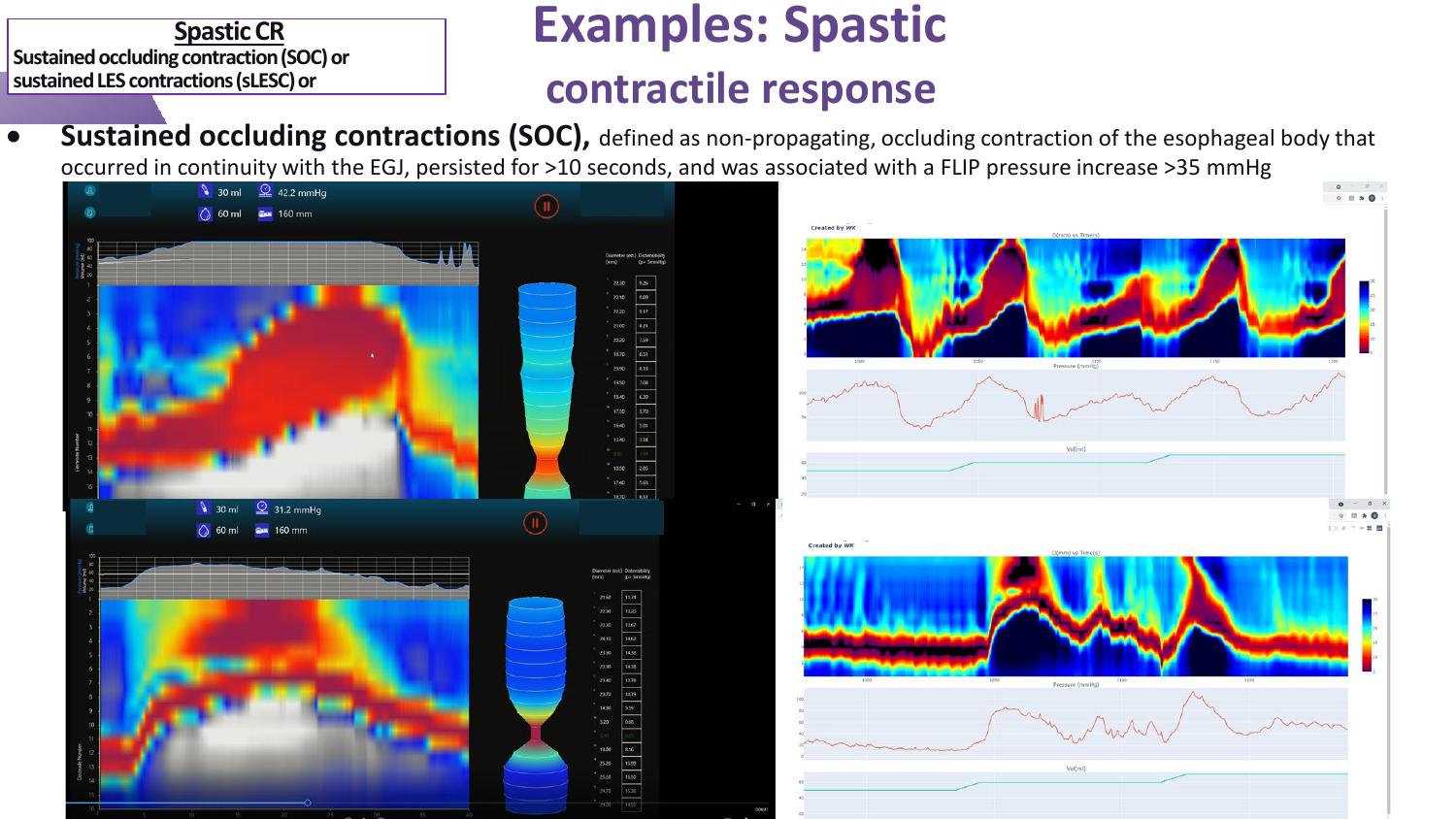


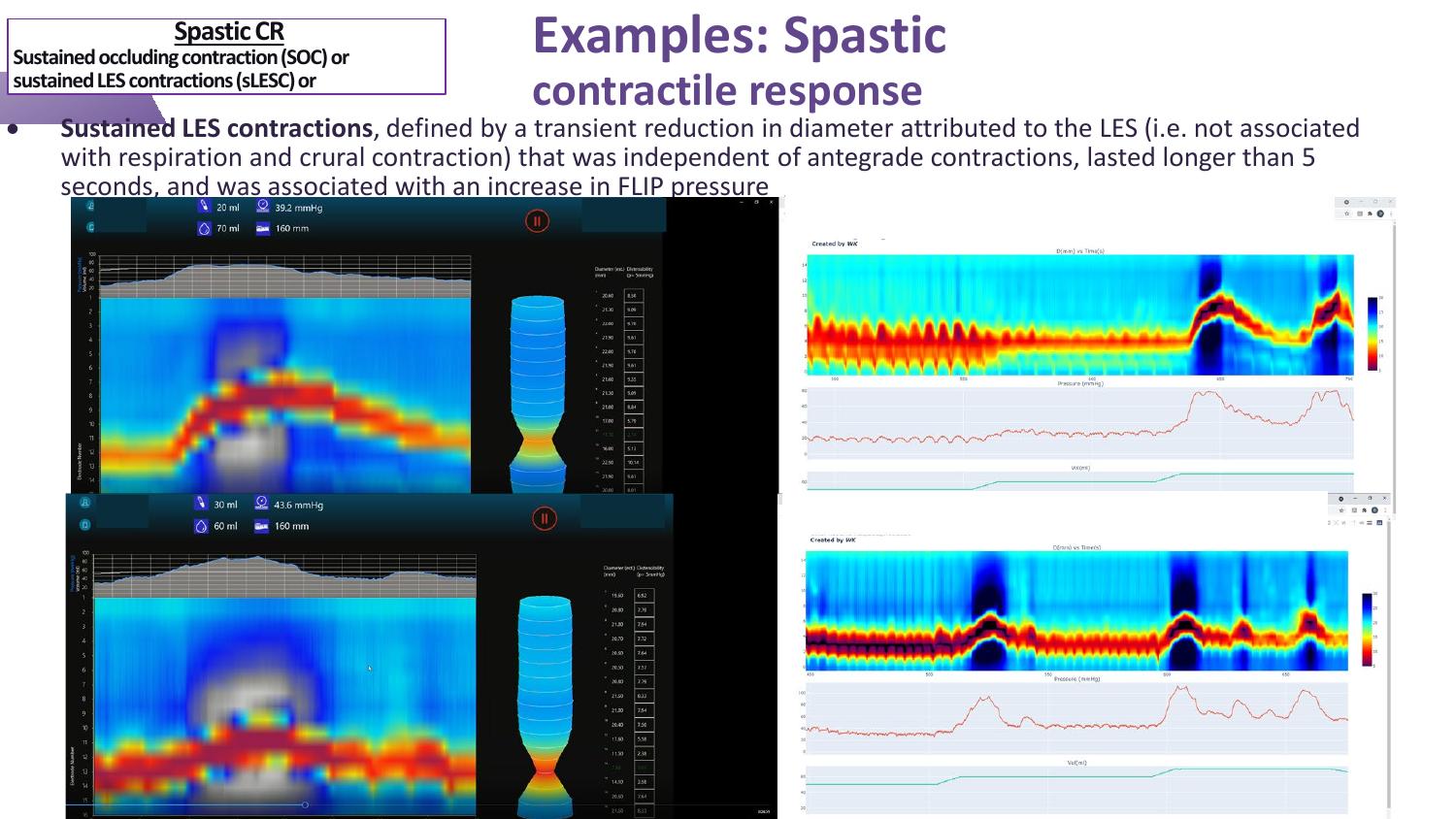


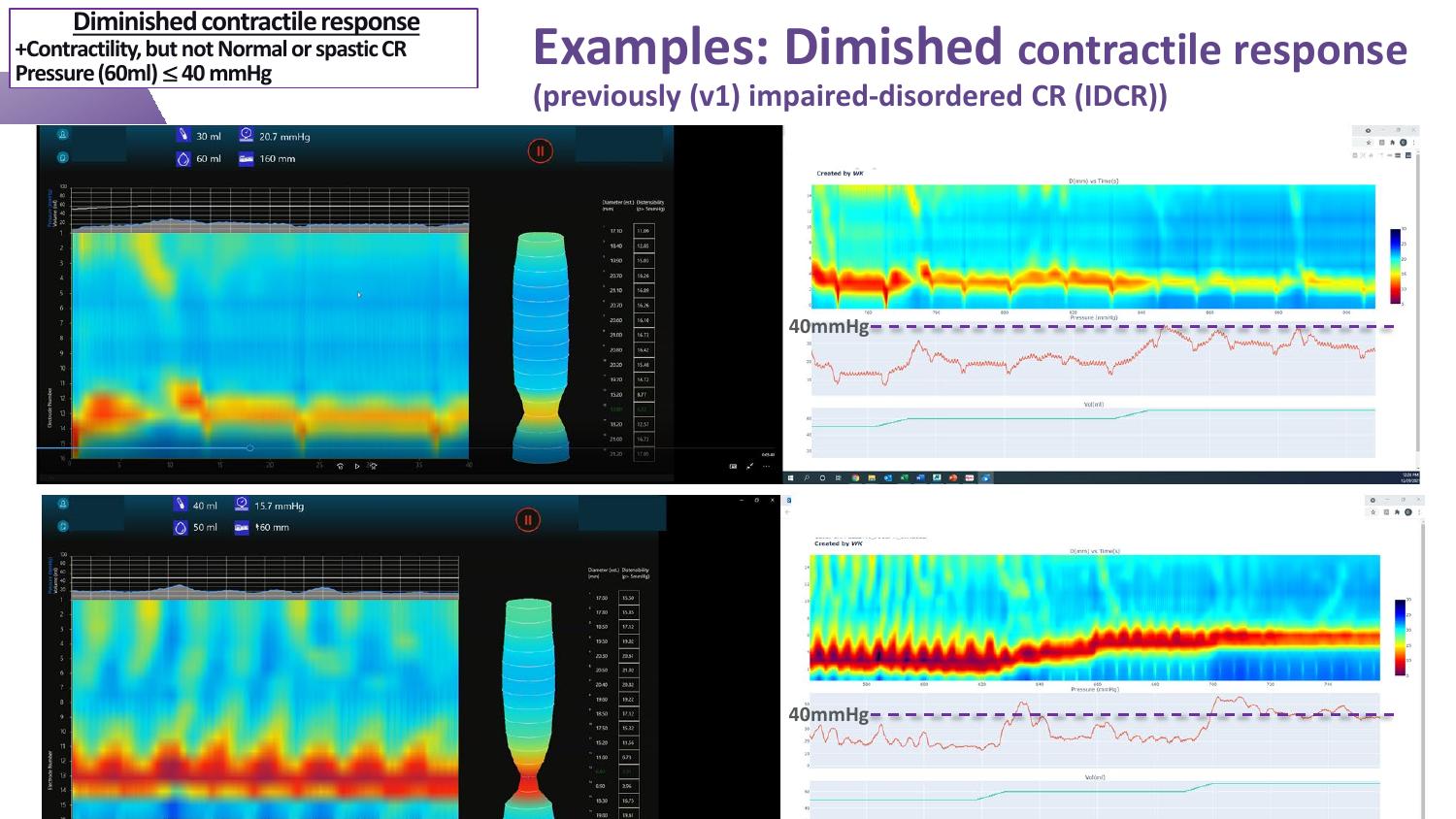


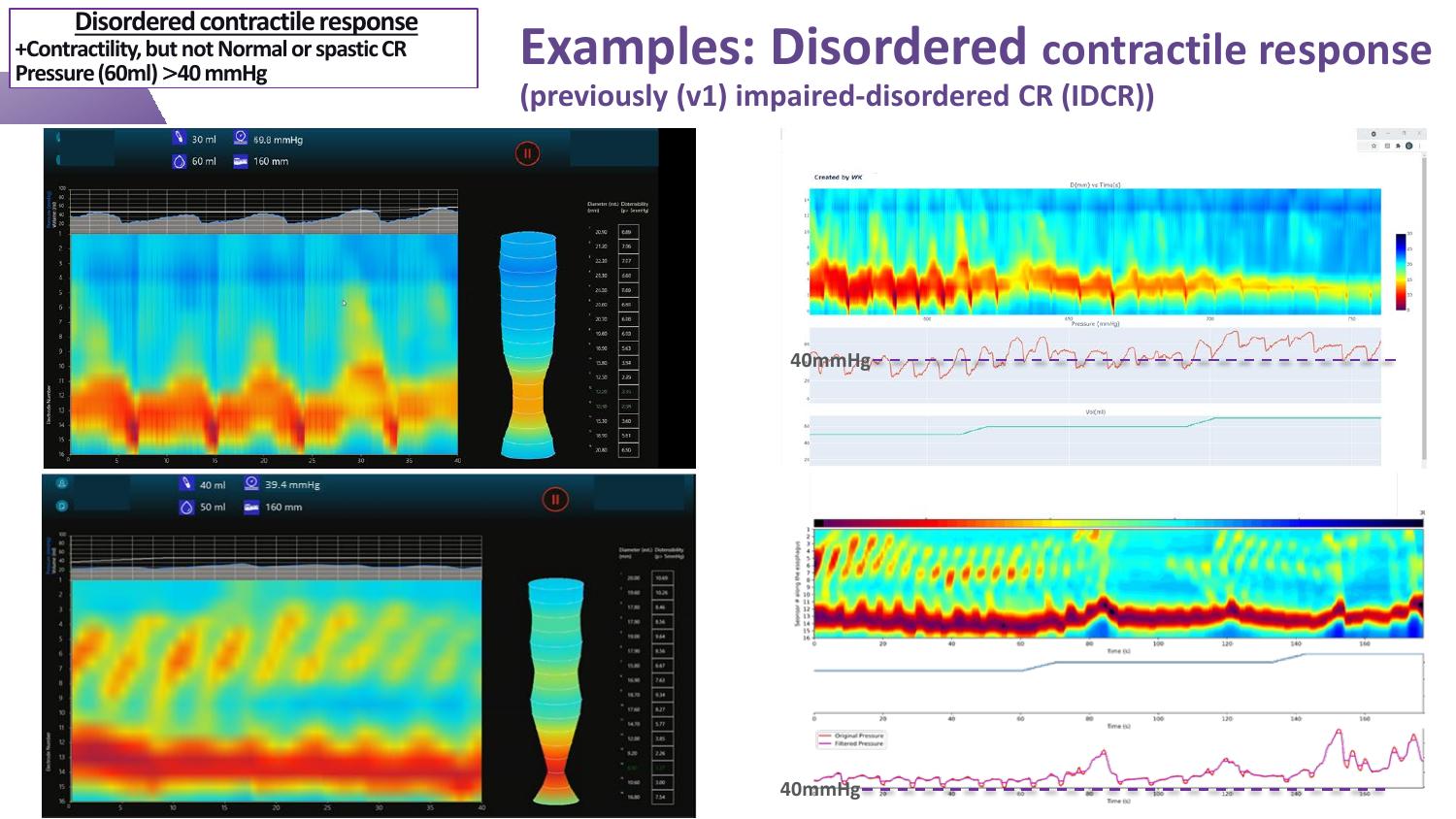


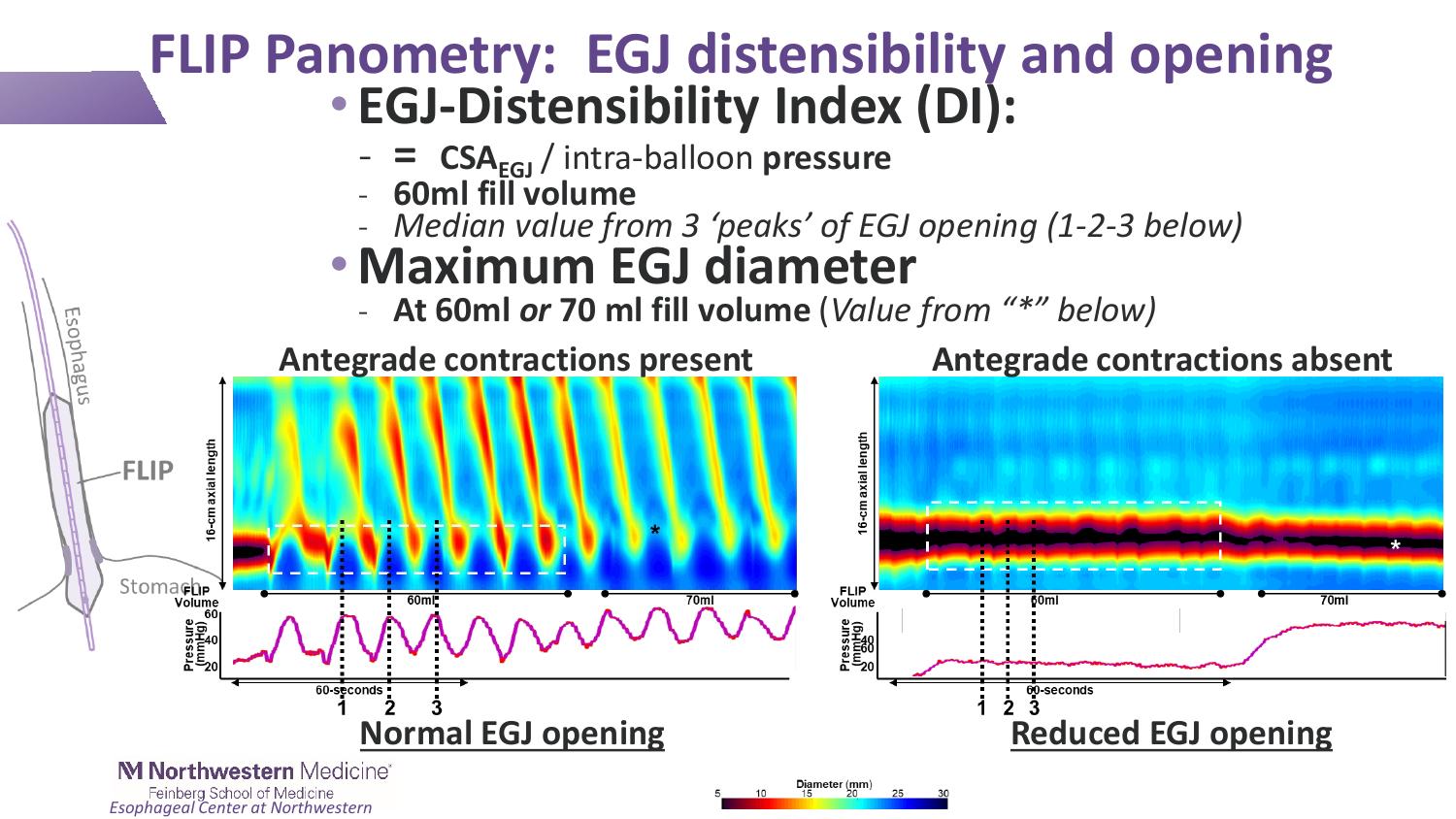


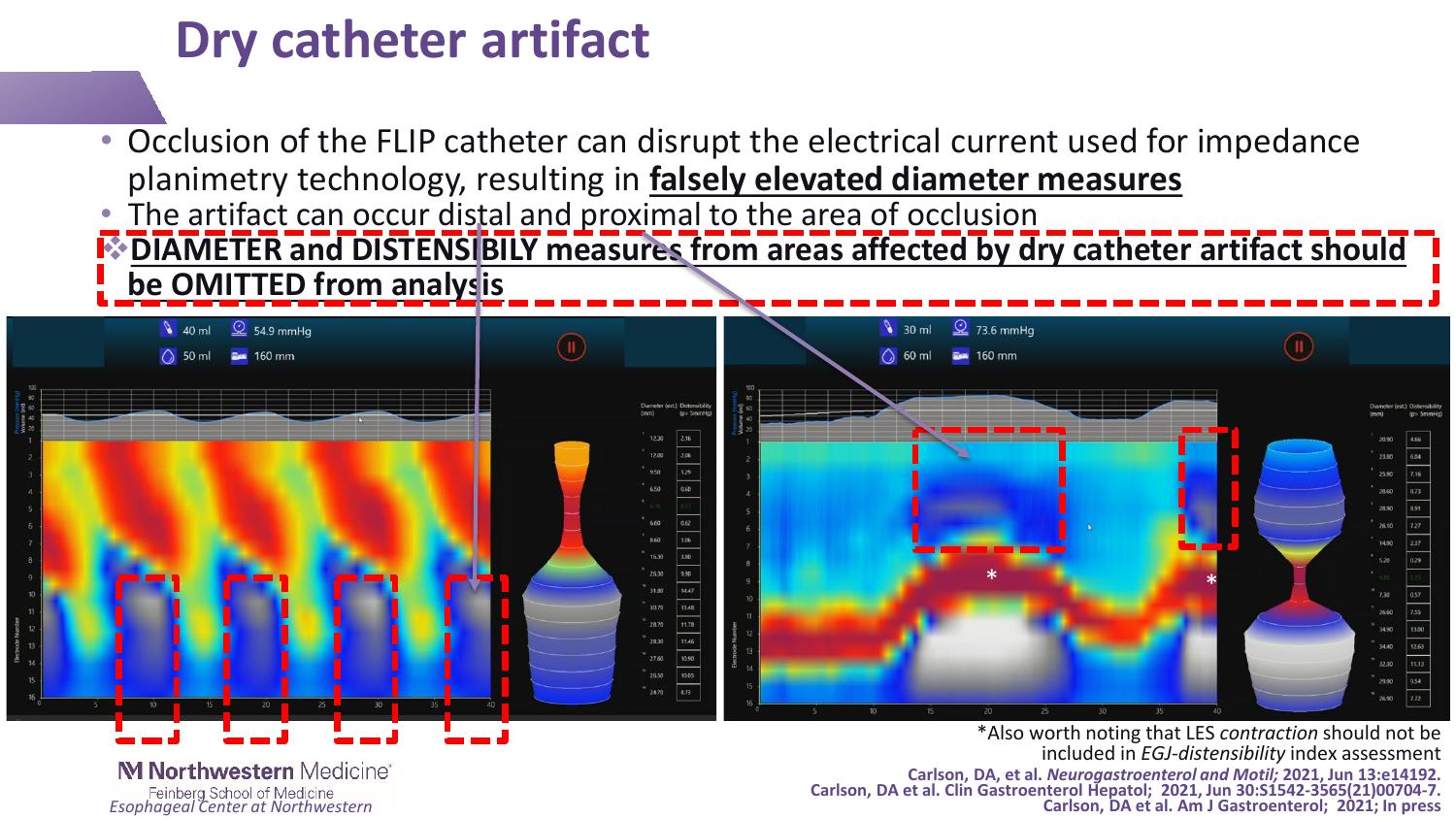


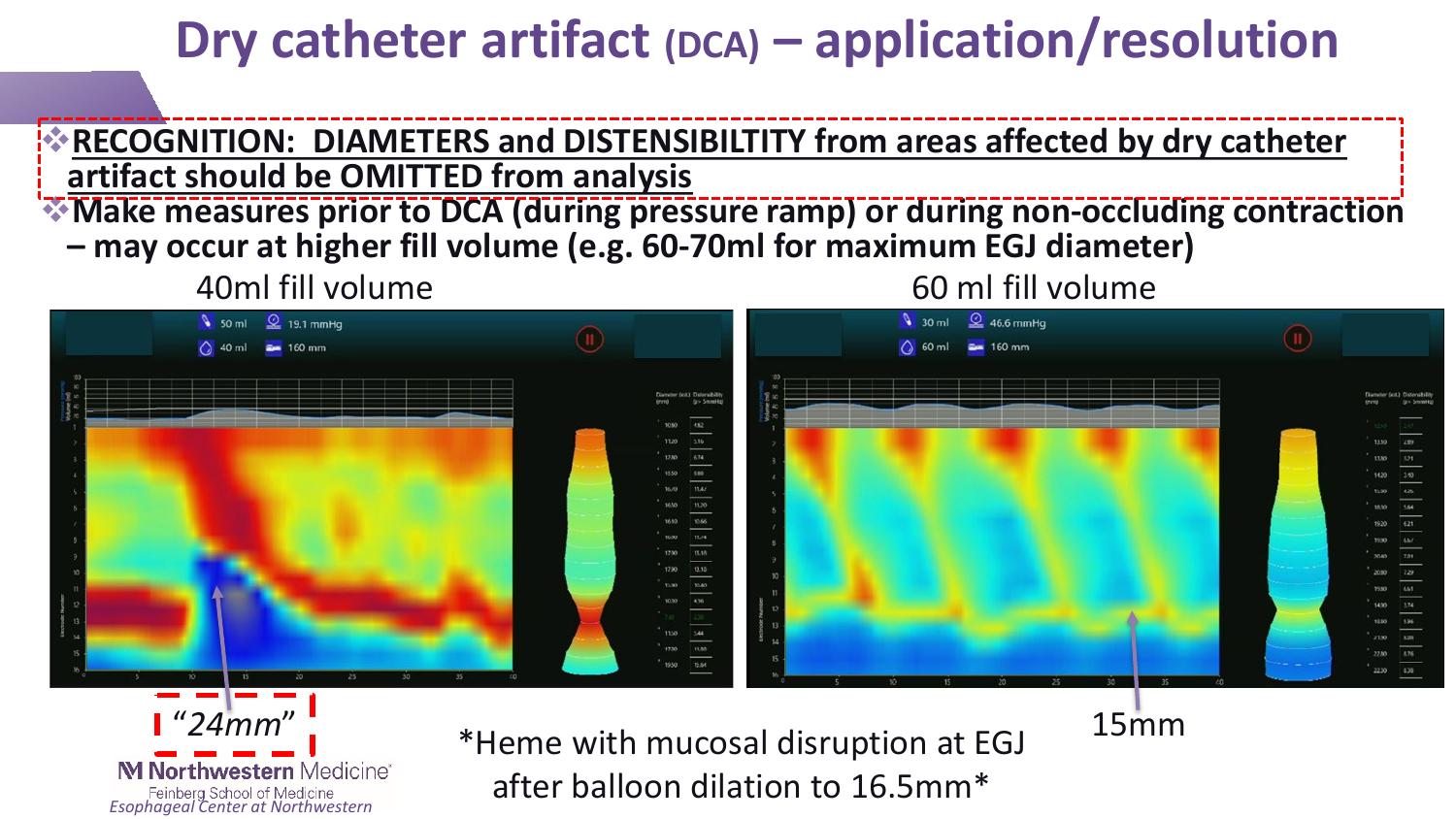


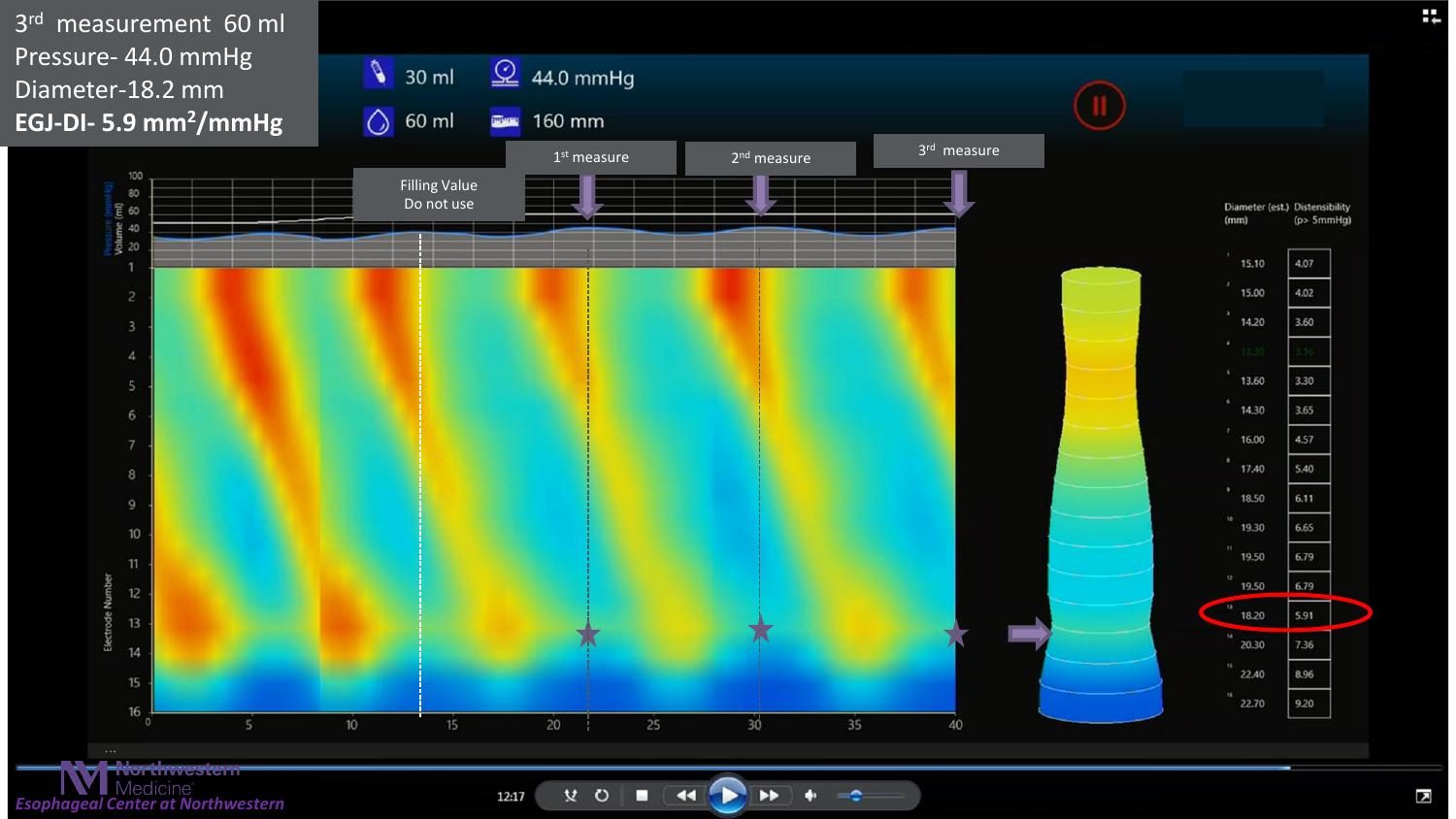


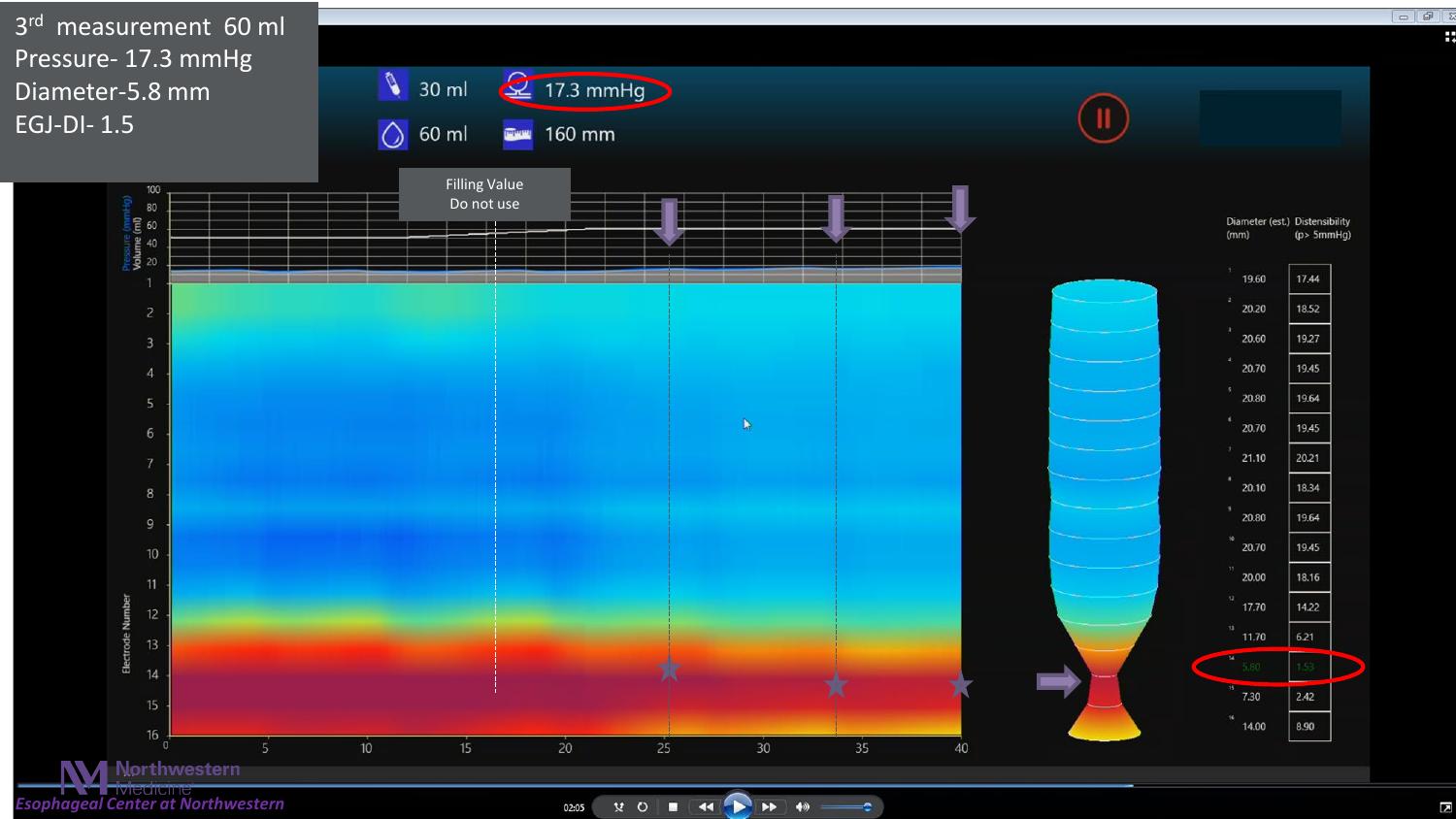


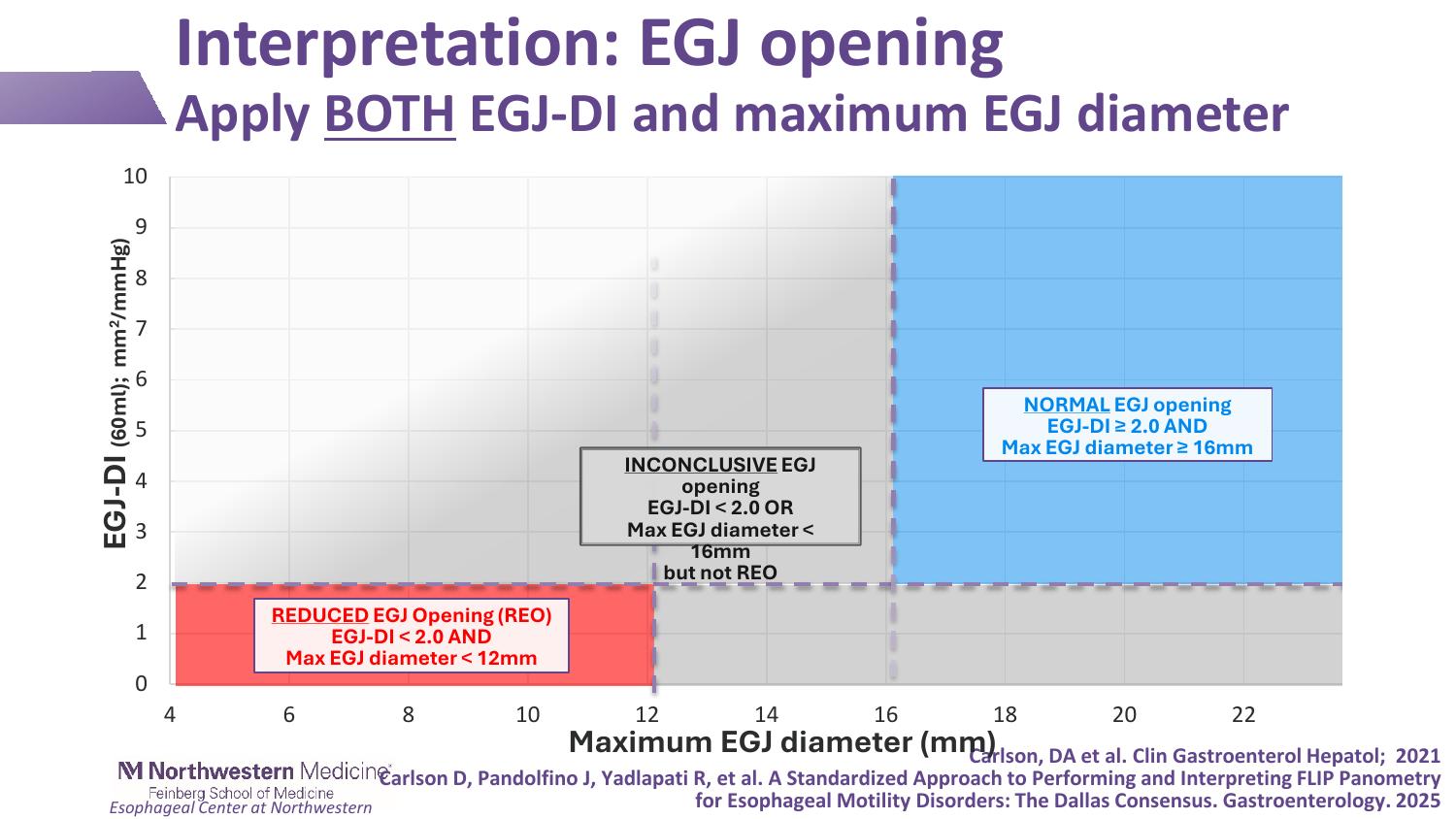


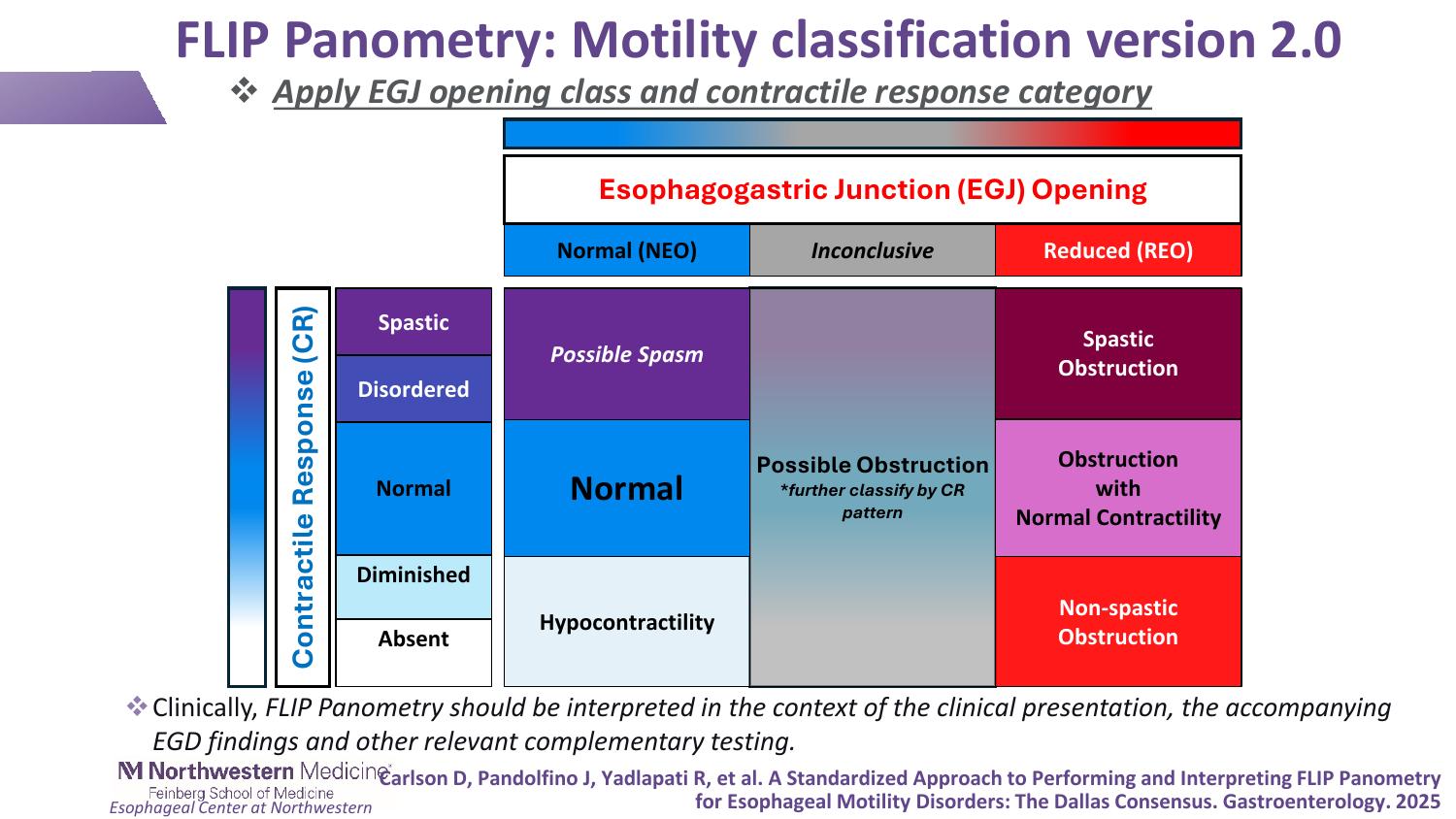


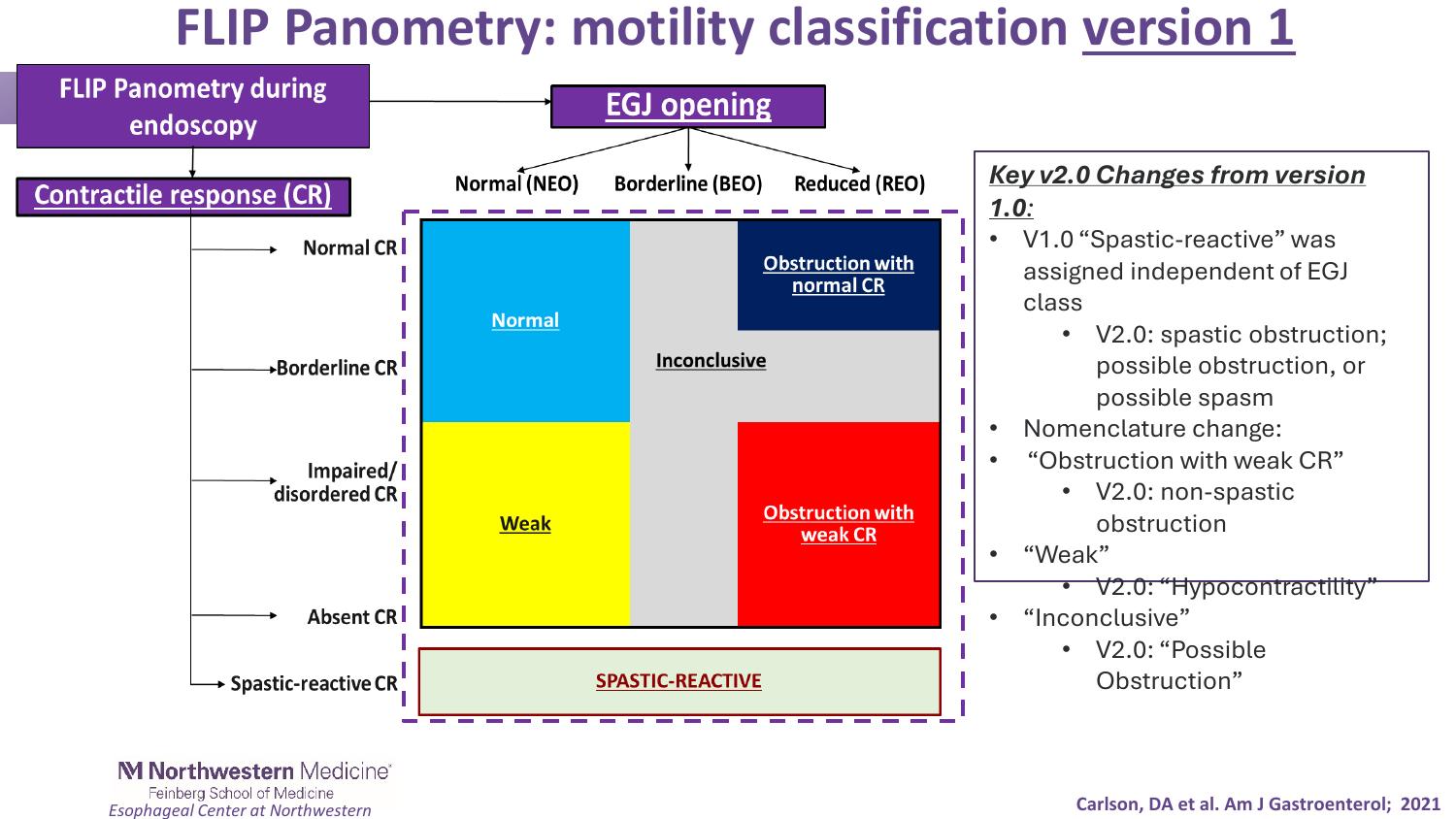


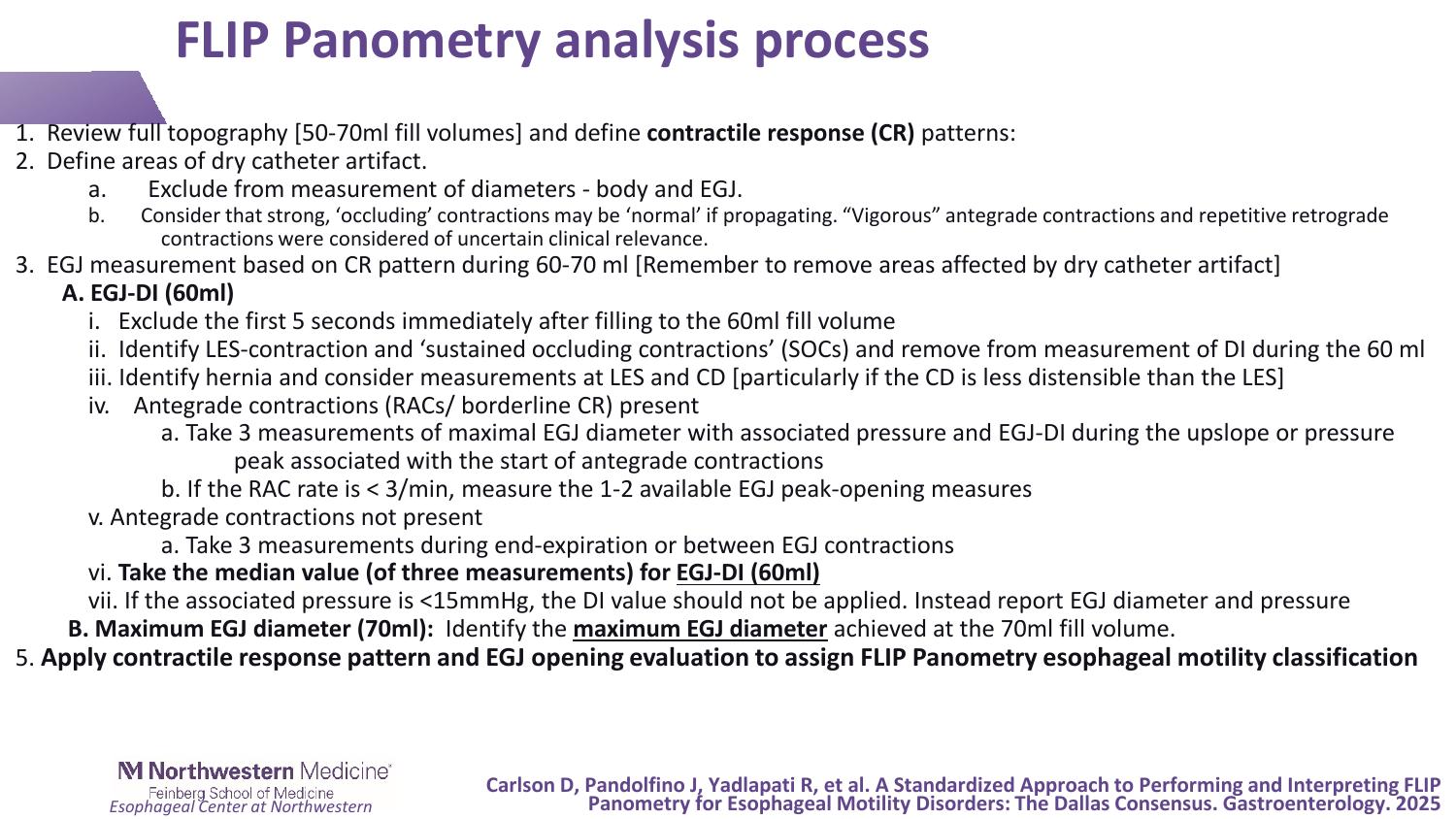


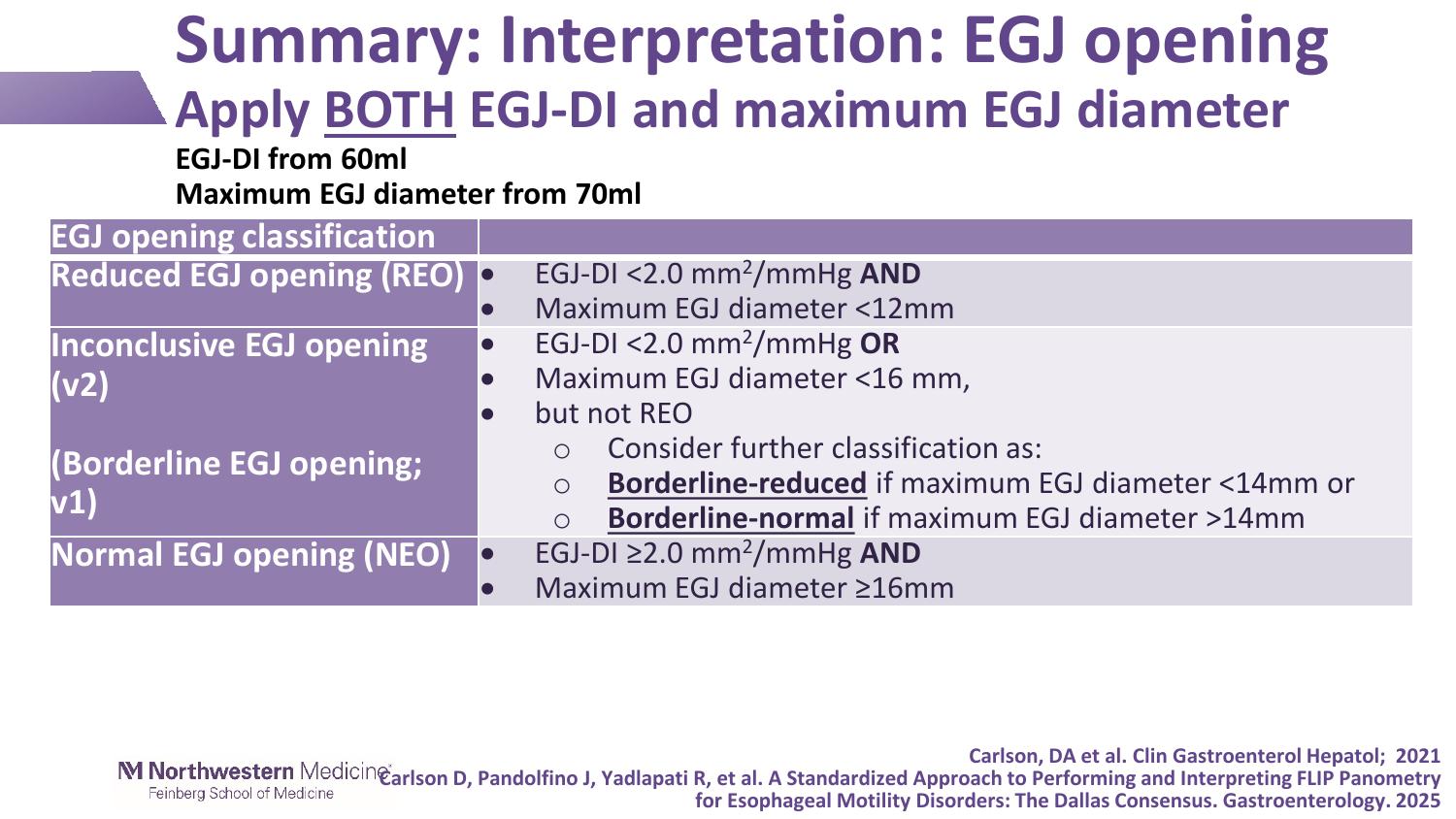


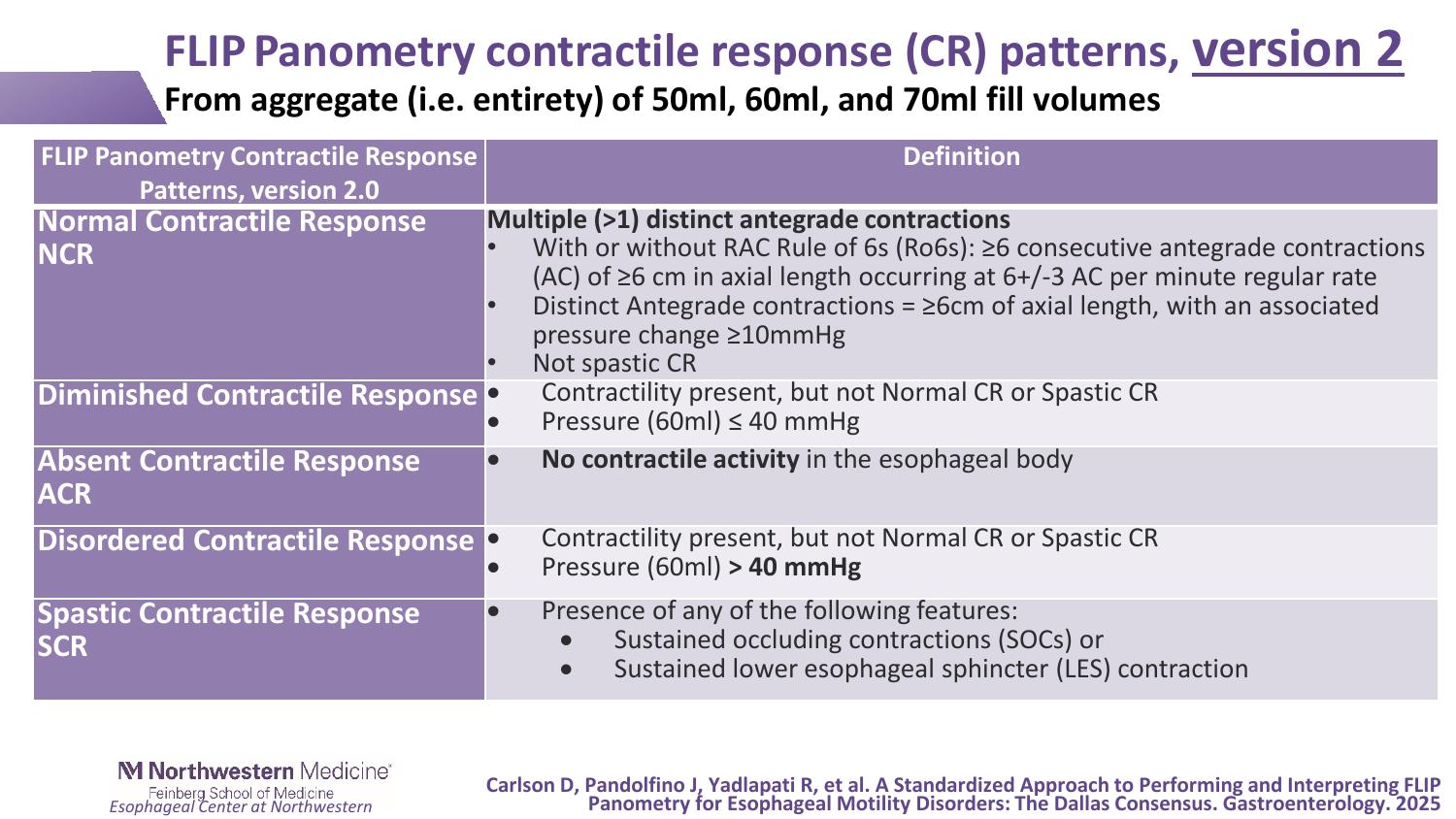


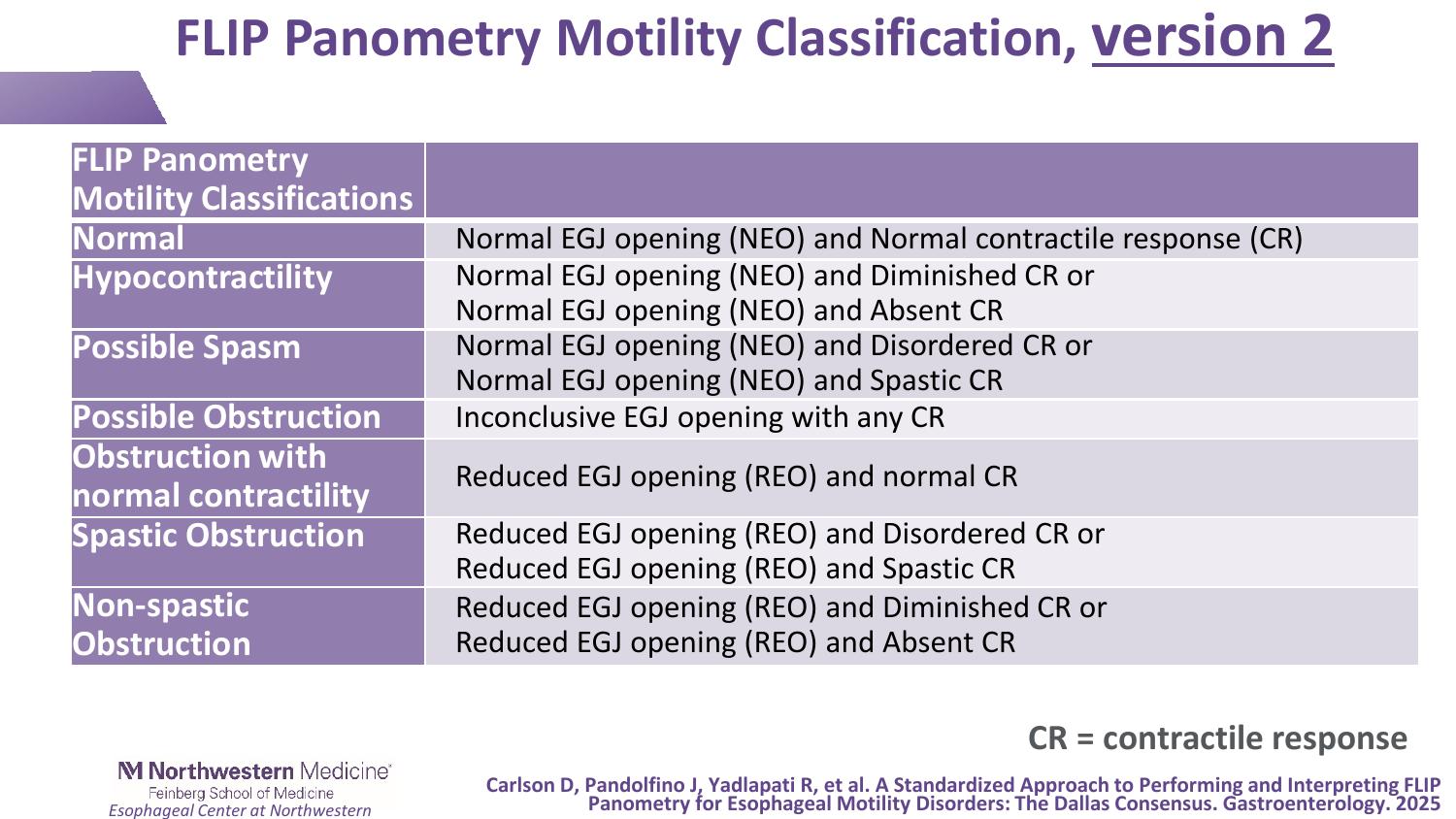


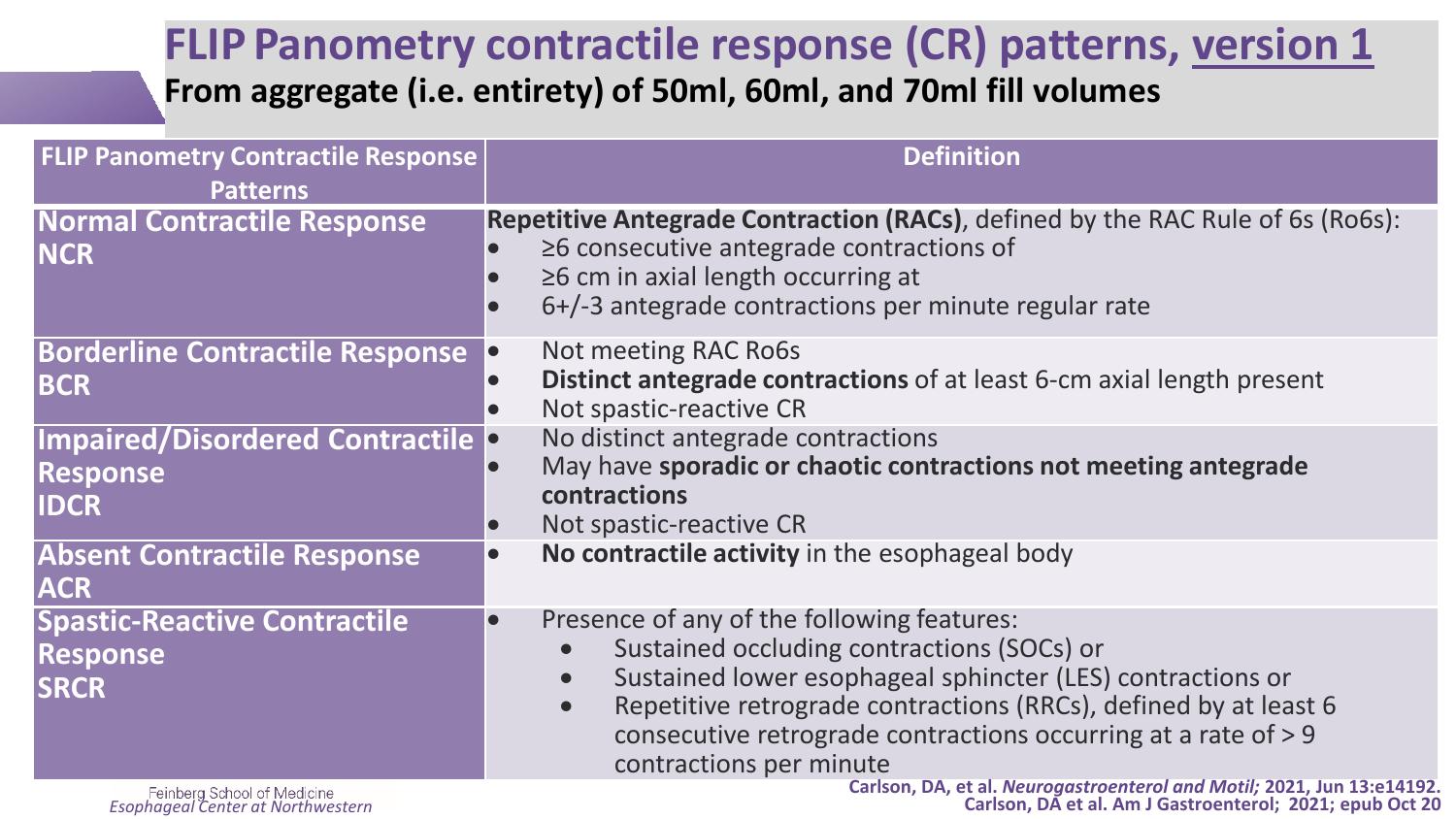


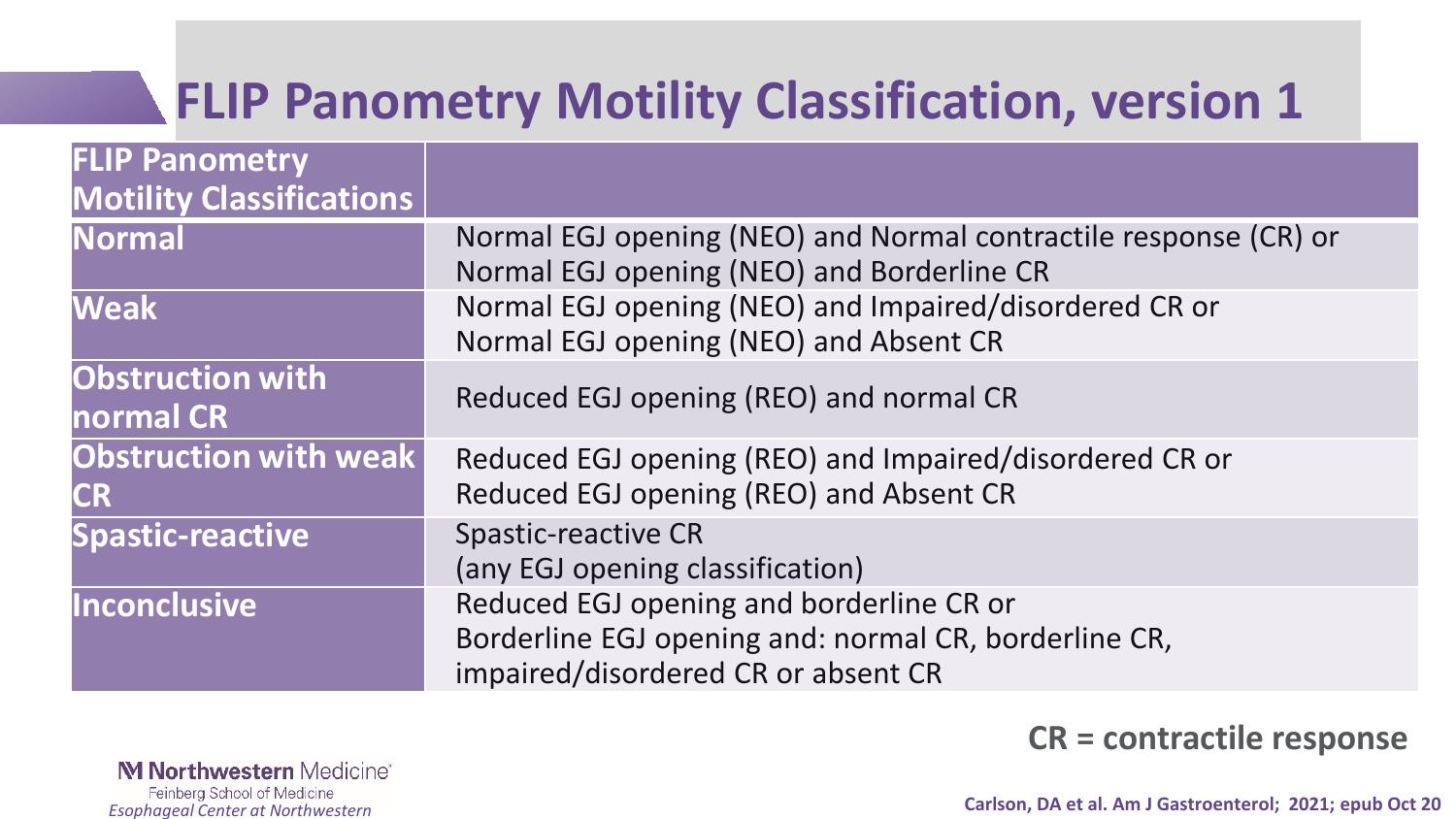


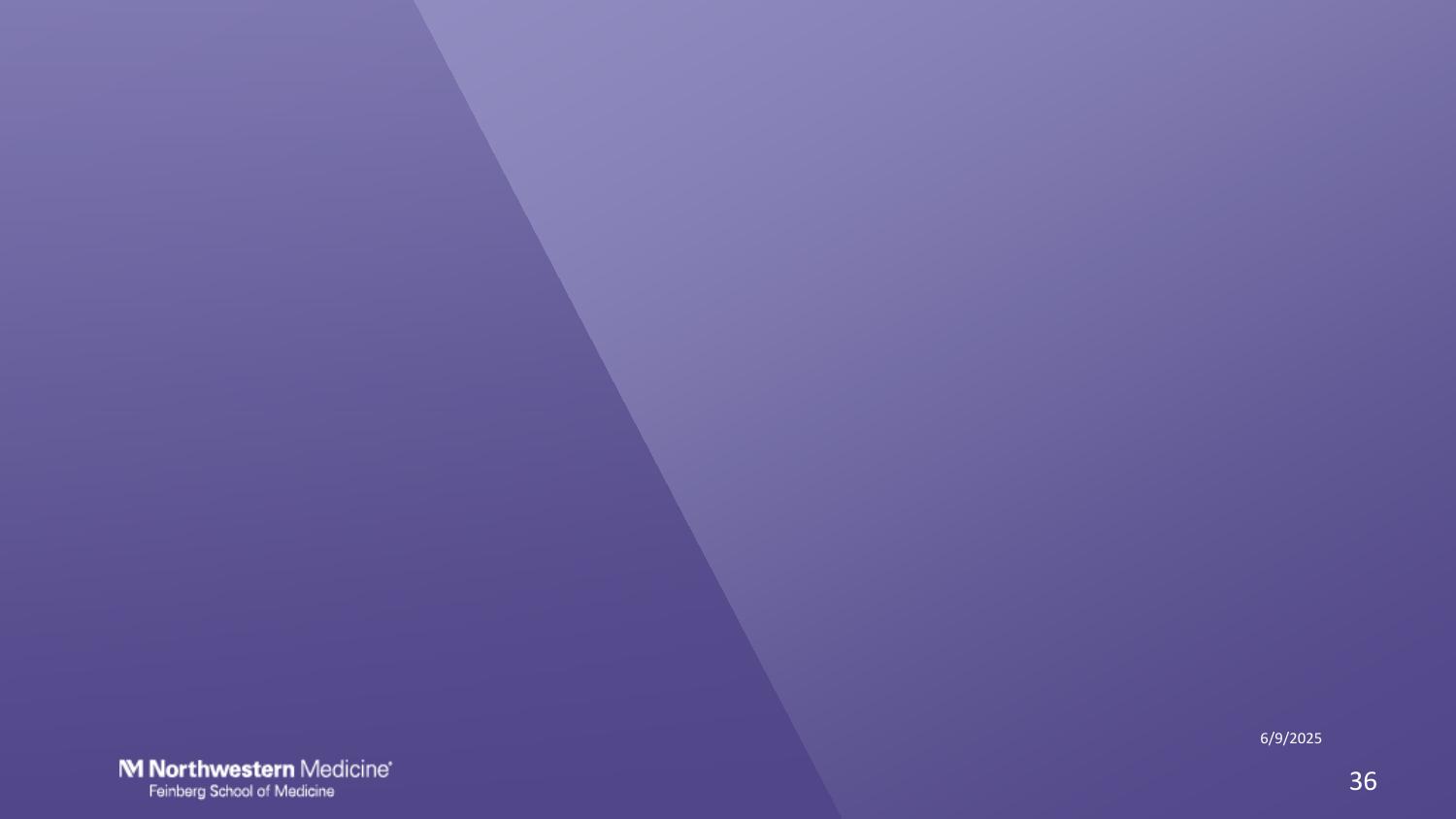

Supplement: Supplementary file 1 — File S1: Slides in FLIP interpretation video tutorial. The brief training of novice raters included a 20‐min instructional tutorial with the following slides. The tutorial covered the concept of FLIP panometry, interpretation using the v1.0 and v2.0 classification schemes, and key differences between the schemes. [file NMO-38-e70386-s003.docx]
