# Supplementary material for: Accuracy of Novice Raters for Esophageal Motility Classifications Using Functional Lumen Imaging Probe Panometry
Source: Neurogastroenterol Motil. 2026 Jul 5;38(7):e70386. doi: 10.1111/nmo.70386 (PMC13334194; doi:10.1111/nmo.70386)

Supplementary File S2. Practice cases used for FLIP interpretation training. Following the video tutorial, novice raters interpreted 10 practice studies and then reviewed correct interpretations with an esophageal specialist. The 10 cases were selected to represent a spectrum of FLIP panometry motility patterns and classifications.


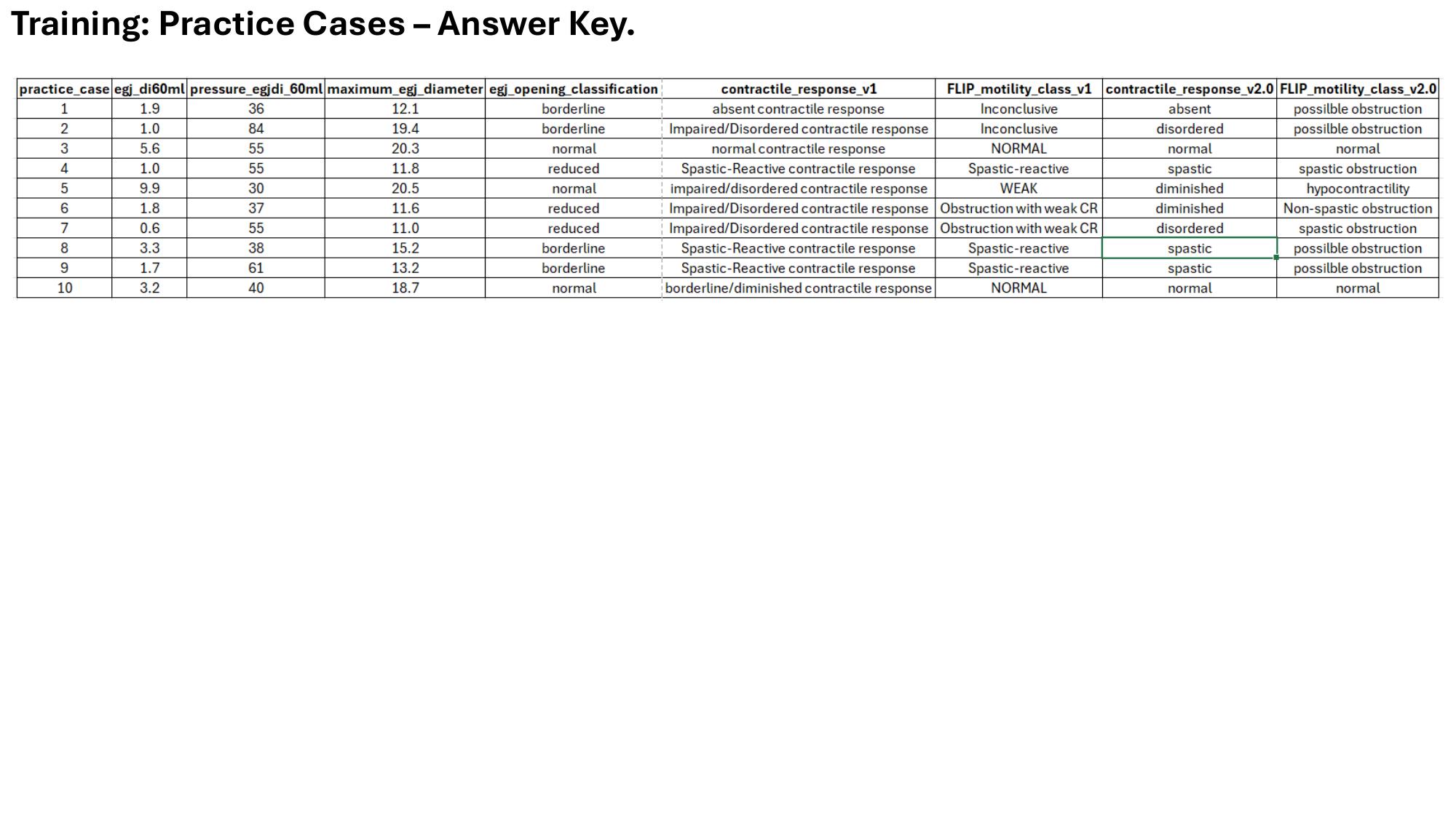


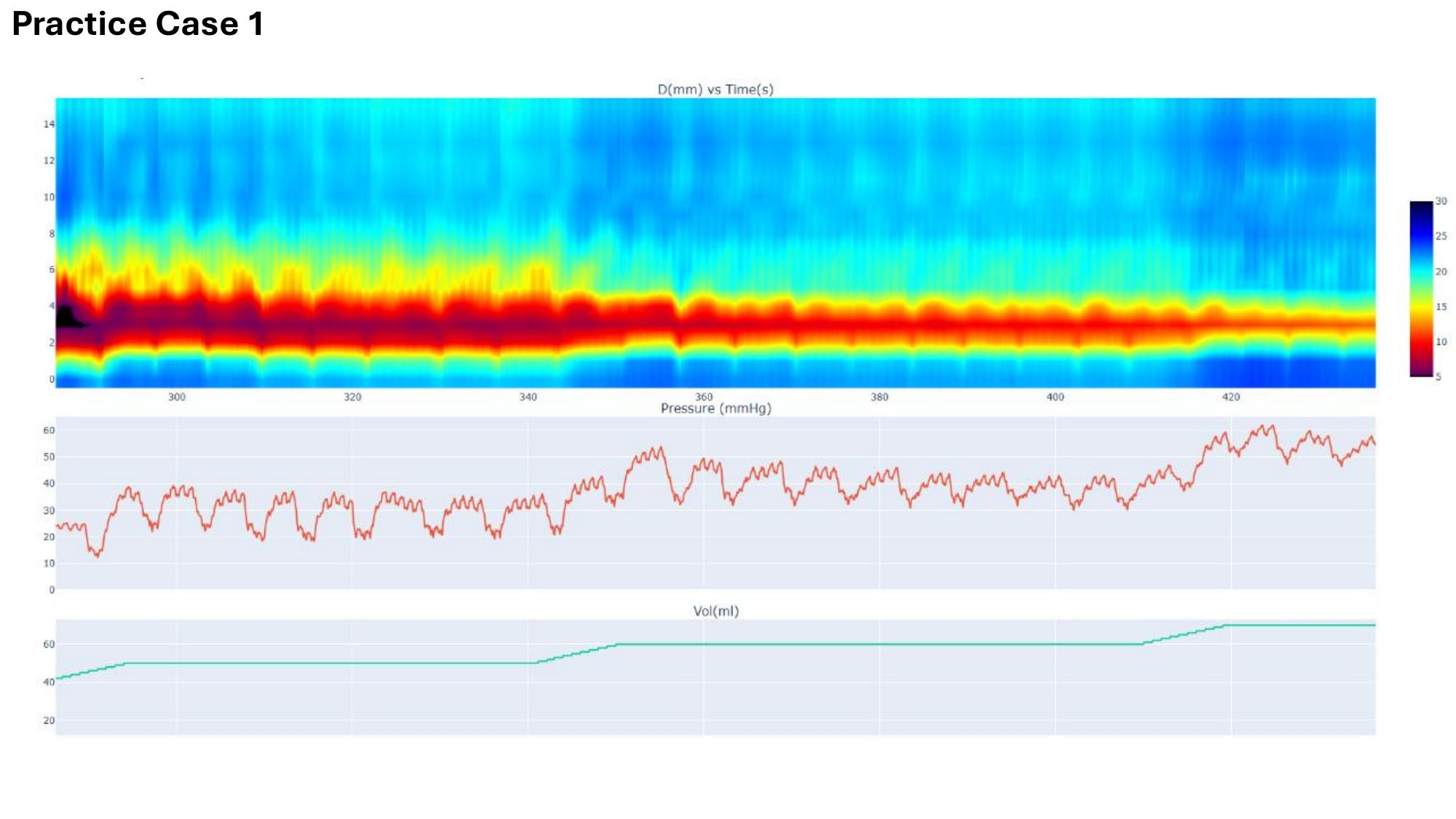


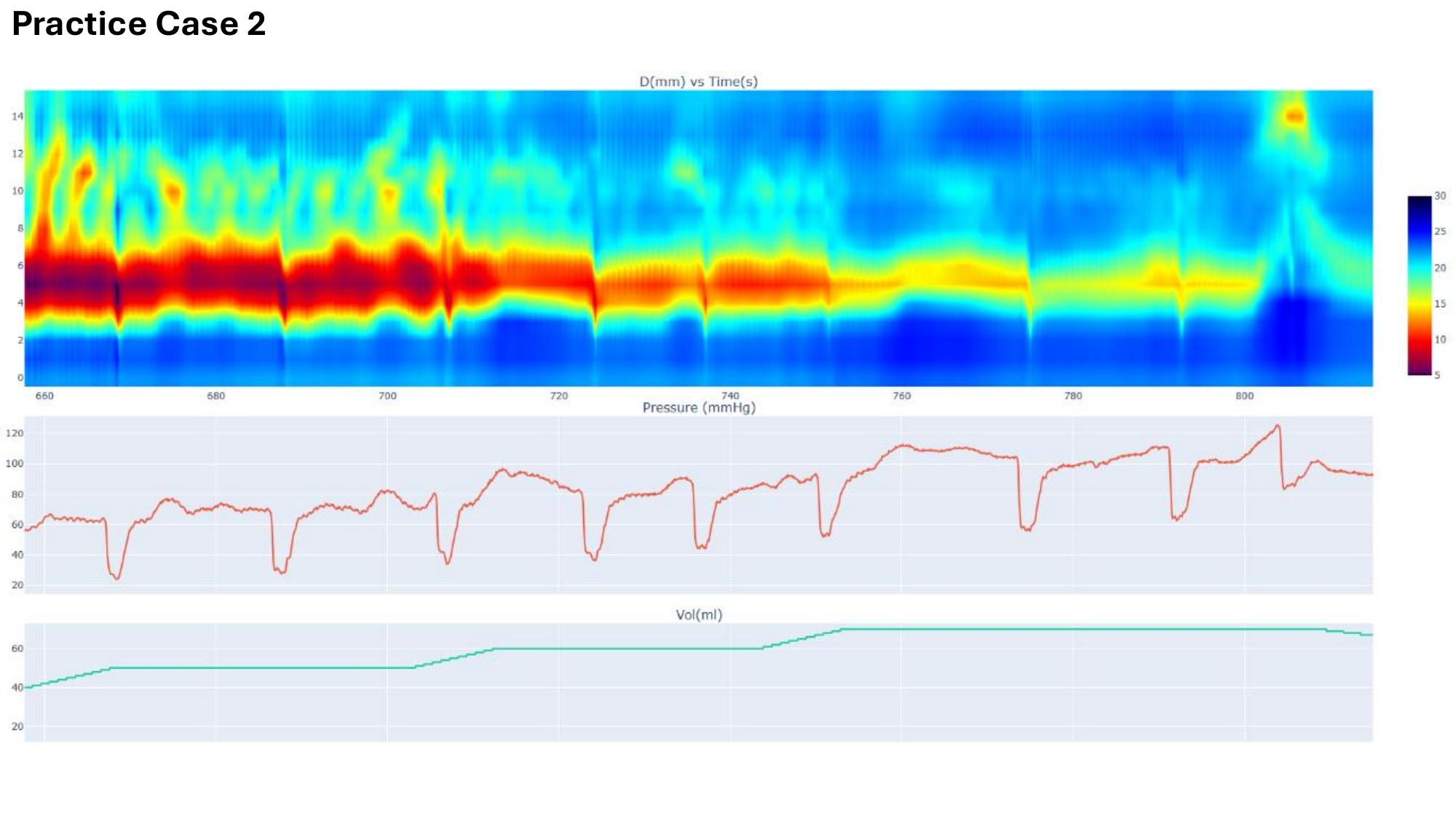


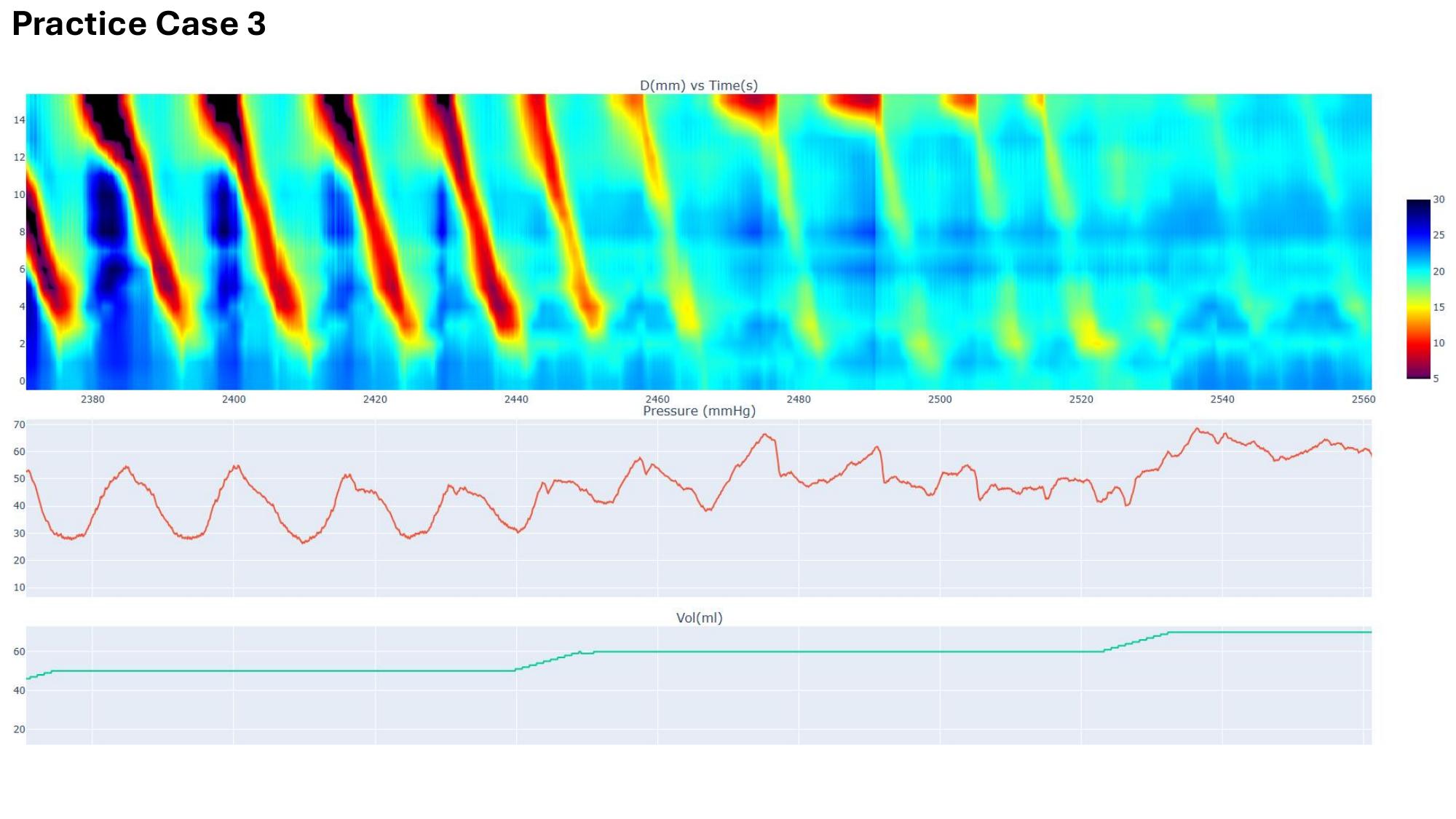


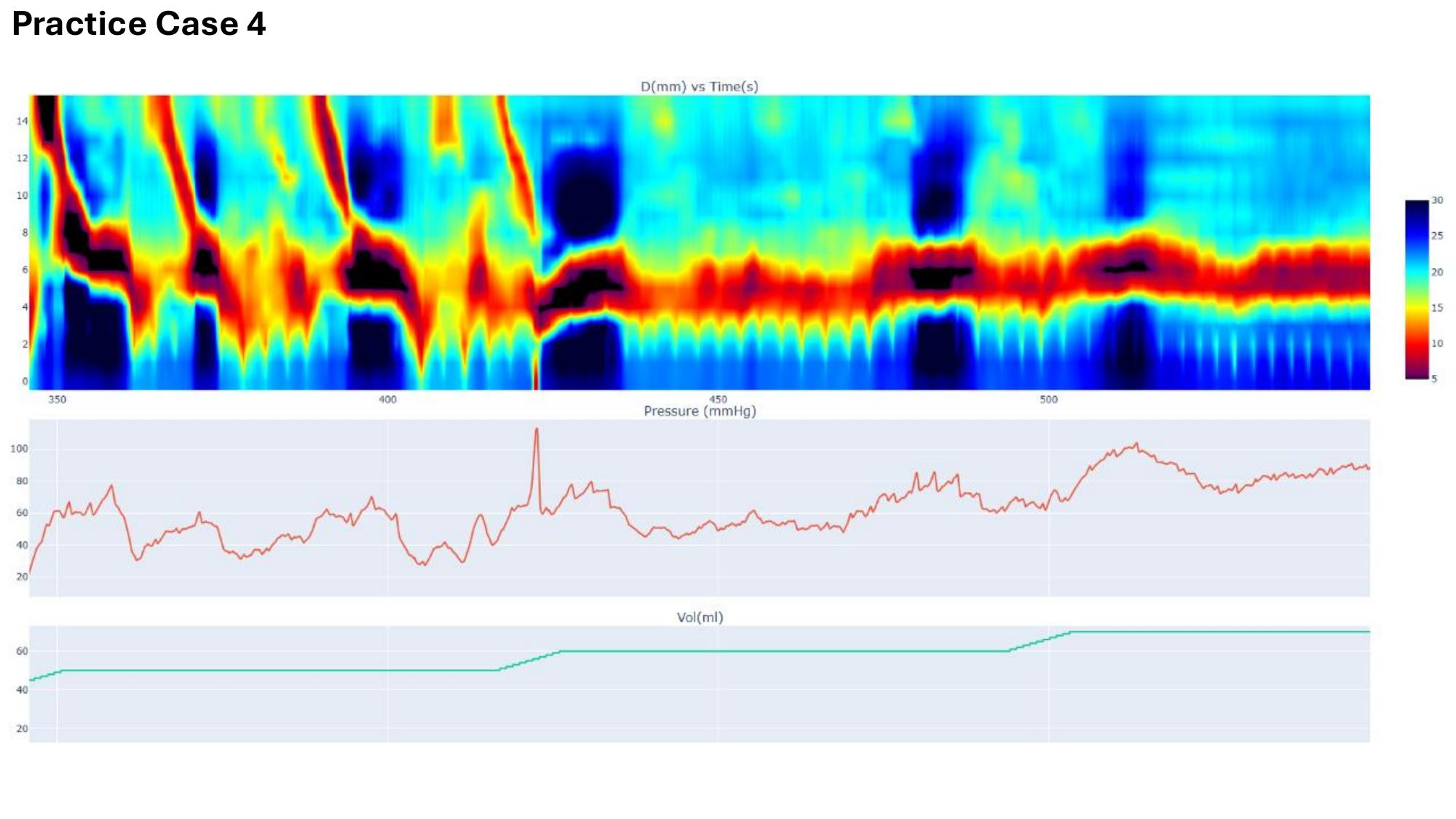


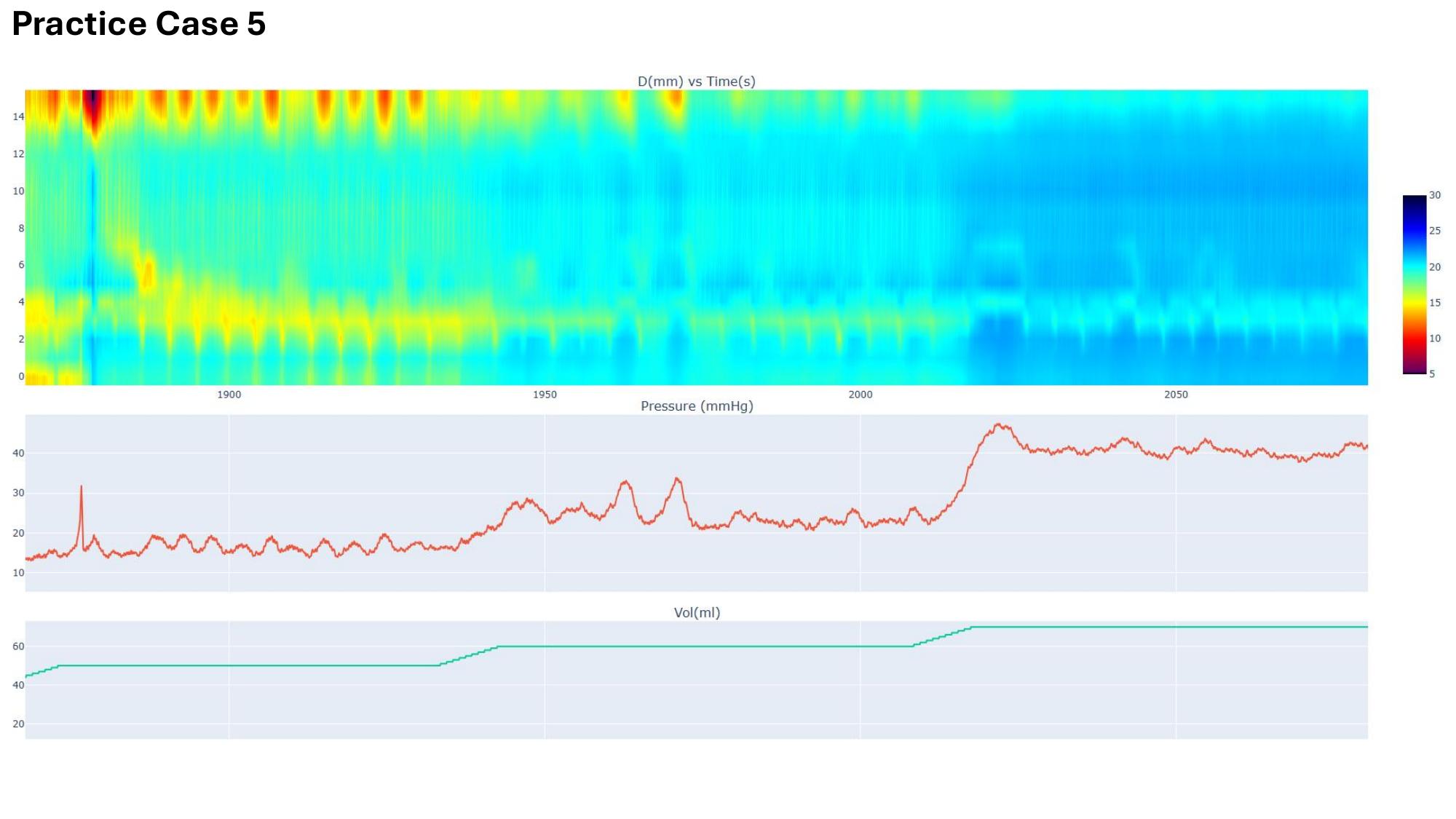


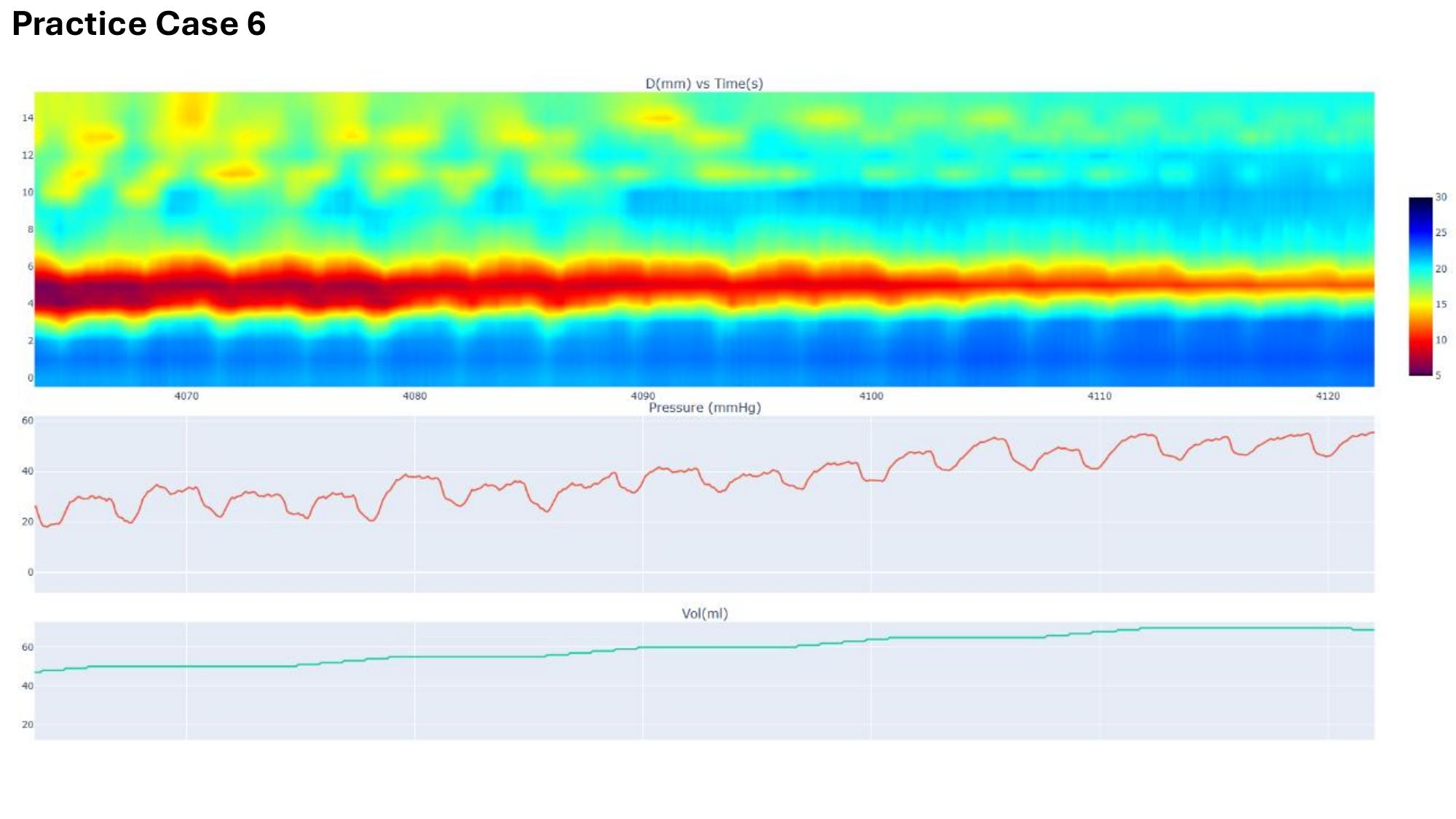


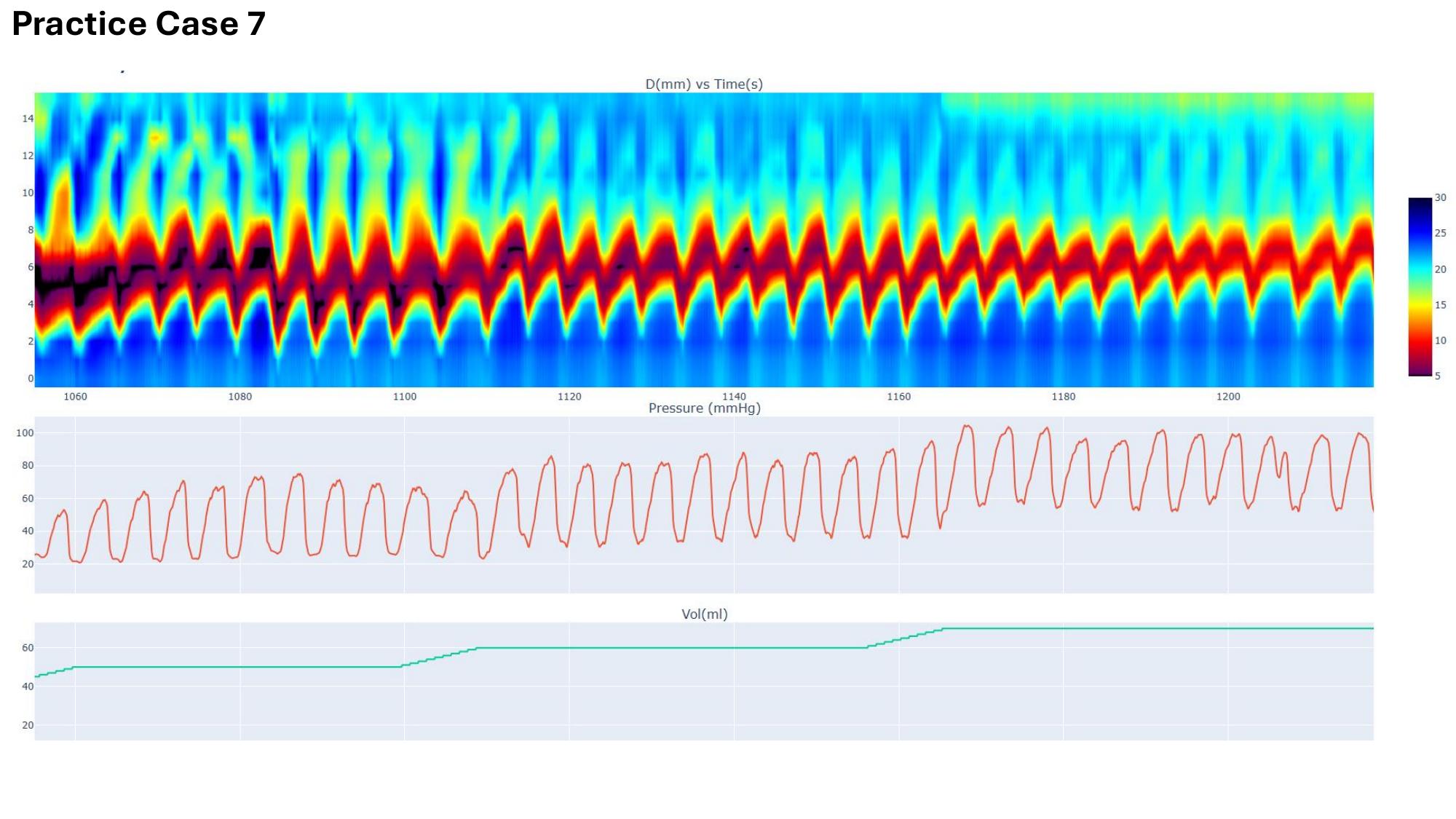


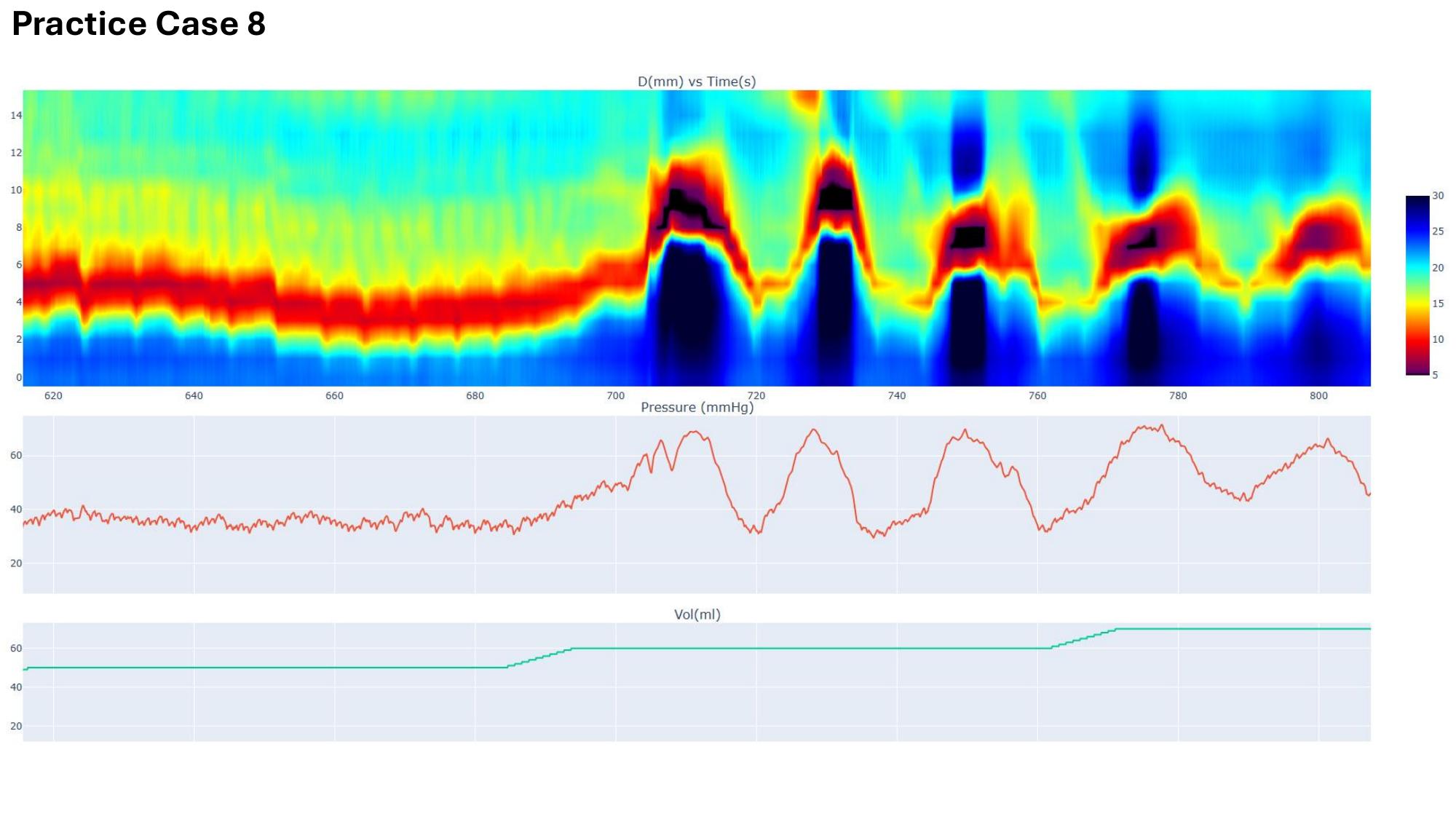


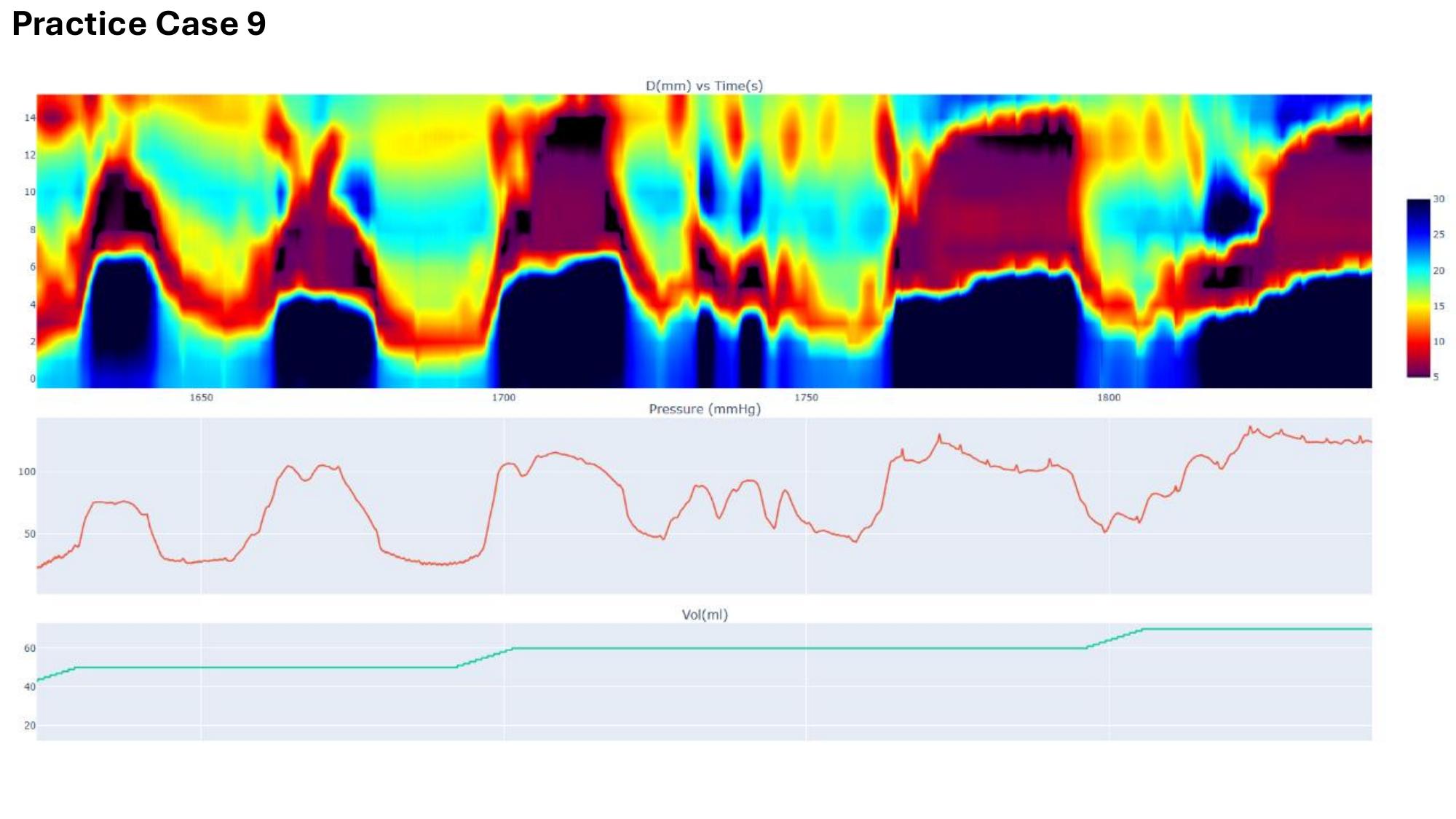


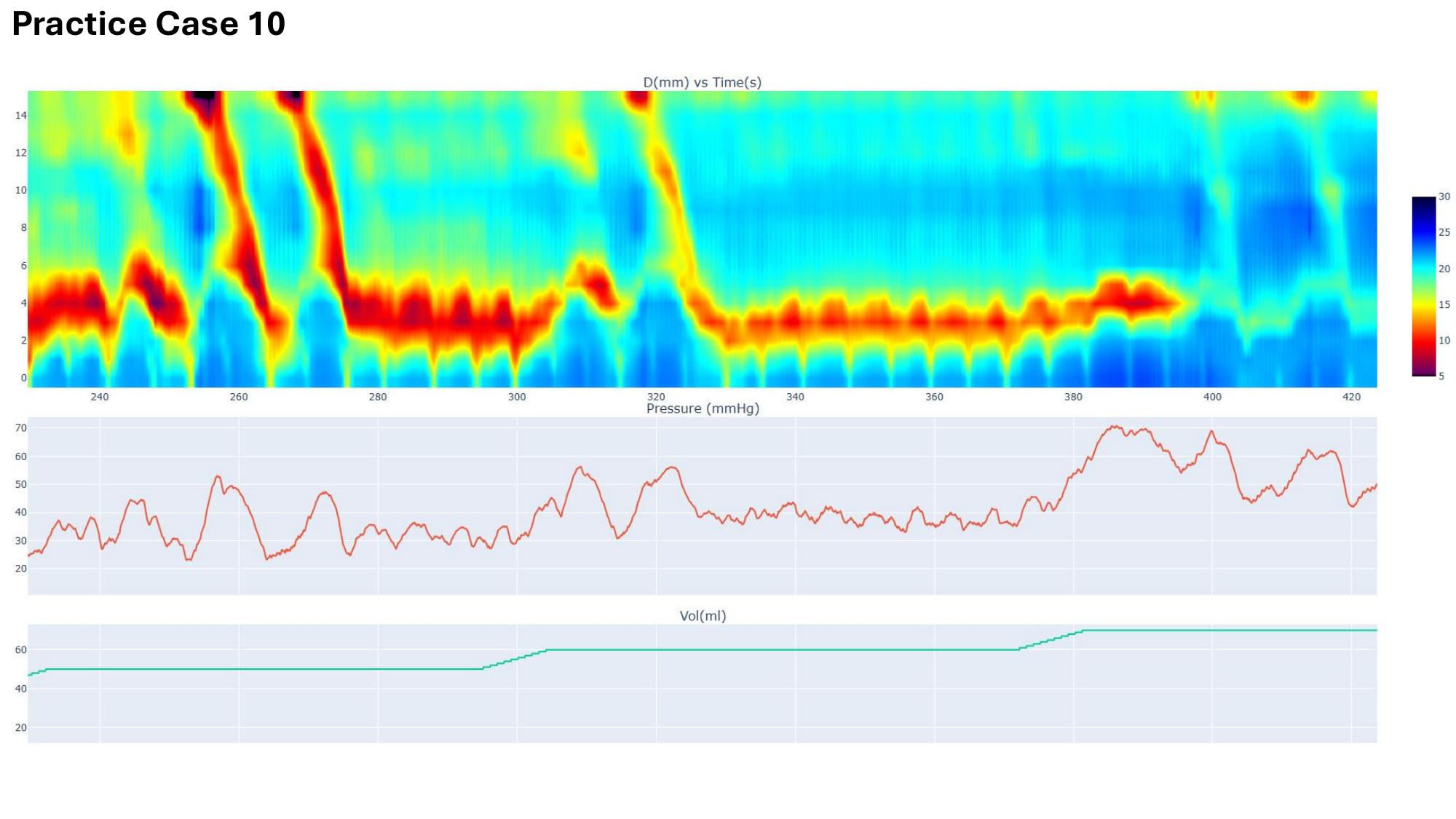

Supplement: Supplementary file 2 — File S2: Practice cases used for FLIP interpretation training. Following the video tutorial, novice raters interpreted 10 practice studies and then reviewed correct interpretations with an esophageal specialist. The 10 cases were selected to represent a spectrum of FLIP panometry motility patterns and classifications. [file NMO-38-e70386-s002.docx]
