# Supplementary material for: Accuracy of Novice Raters for Esophageal Motility Classifications Using Functional Lumen Imaging Probe Panometry
Source: Neurogastroenterol Motil. 2026 Jul 5;38(7):e70386. doi: 10.1111/nmo.70386 (PMC13334194; doi:10.1111/nmo.70386)
Supplement: Supplementary file 3 — Table S1: Case (patient) demographics and characteristics. Raters interpreted FLIP studies of 40 patients evaluated for dysphagia at the Esophageal Center of Northwestern. Patients with previous foregut surgery, mechanical obstruction, or abnormal anatomy were excluded from the study due to risk of secondary esophageal dysmotility. Raters were informed that all patients presented with dysphagia, had no prior foregut surgery, and had no evidence of mechanical obstruction on upper endoscopy. [file NMO-38-e70386-s001.docx]

Supplementary Table S1. Case (patient) demographics and characteristics. Raters interpreted FLIP studies of 40 patients evaluated for dysphagia at the Esophageal Center of Northwestern. Patients with previous foregut surgery, mechanical obstruction, or abnormal anatomy were excluded from the study due to risk of secondary esophageal dysmotility. Raters were informed that all patients presented with dysphagia, had no prior foregut surgery, and had no evidence of mechanical obstruction on upper endoscopy.

|  |  |
| --- | --- |
| **Age, years; mean (SD)** | 53 (15) |
| **Sex, n (%) female** | 23 (58) |
| **Primary Indication**^†^**, n (%)**  **Dysphagia**  **Chest Pain**  **Reflux-symptoms** | 34 (85)  1 (2)  5 (13) |
| **EGD findings** |  |
| **Erosive Esophagitis, n (%)**  **None**  **Los-Angeles A**  **Los-Angeles B** | 39 (98)  1 (2)  0 |
| **Hiatal Hernia, n (%)**  **None**  **Small (1-3cm)** | 30 (75)  10 (25) |
| **High-resolution manometry / Chicago Classification v4.0, n (%)** |  |
| **Type I achalasia**  **Type II achalasia**  **Type III achalasia**  **EGJ outflow obstruction**  **Absent contractility**  **Ineffective Esophageal Motility**  **Normal motility** | 4 (10)  7 (18)  4 (10)  6 (15)  3 (8)  3 (8)  13 (33) |

^†^All patients also reported dysphagia.
